# Disentangling wealth effects on fertility in 64 low- and middle-income countries

**DOI:** 10.1017/ehs.2020.62

**Published:** 2020-11-23

**Authors:** Joseph Hackman, Daniel Hruschka

**Affiliations:** 1 University of Utah, Department of Anthropology, Salt Lake City, Utah, USA; 2 Arizona State University, School of Human Evolution and Social Change, Arizona, USA

**Keywords:** Fertility transition, wealth, status, wealth index, embodied capital

## Abstract

Studies have shown mixed associations between wealth and fertility, a finding that has posed ongoing puzzles for evolutionary theories of human reproduction. However, measures of wealth do not simply capture economic capacity, which is expected to increase fertility. They can also serve as a proxy for market opportunities available to a household, which may reduce fertility. The multifaceted meaning of many wealth measures obscures our ability to draw inferences about the relationship between wealth and fertility. Here, we disentangle economic capacity and market opportunities using wealth measures that do not carry the same market-oriented biases as commonly used asset-based measures. Using measures of agricultural and market-based wealth for 562,324 women across 111,724 sampling clusters from 151 DHS surveys in 64 countries, we employ a latent variable structural equation model to estimate (a) latent variables capturing economic capacity and market opportunity and (b) their effects on completed fertility. Market opportunities had a consistent negative effect on fertility, while economic capacity had a weaker but generally positive effect on fertility. The results show that the confusion between operational measures of wealth and the concepts of economic capacity can impede our understanding of how material resources and market contexts shape reproduction.

## Graphical abstract

**Figure:**
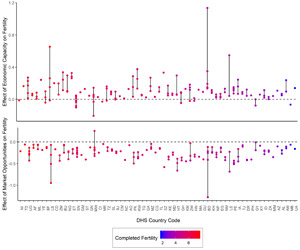

**Media summary:** Multiple measures of wealth can parse the effects of economic capacity from market opportunities on fertility in LMICS.

## Introduction

### The association between socioeconomic status and fertility

The global transition to low fertility in the midst of modernisation has been a biological puzzle for decades (Handwerker, 1986; Kaplan, 1996; Vining, 1986). A central feature of this puzzle is the inconsistent relationship between socioeconomic status and fertility across human contexts. Studies in anthropology have often documented positive effects of wealth and status on reproduction and fertility across a range of traditional, small-scale and subsistence populations (Cronk, 1991; Flinn, 1986; Turke & Betzig, 1985). For example, when wealth is measured as food energy, researchers have often found a strong positive association between wealth and reproductive success (Borgerhoff Mulder & Beheim, 2011; Kaplan, Lancaster, Tucker, & Anderson, 2002). When wealth comes in the form of material assets in these contexts, there is also a positive association with reproductive output, particularly for men (Borgerhoff Mulder & Beheim, 2011; Cronk, 1991; Flinn, 1986; Nettle & Pollet, 2008). These studies suggest that in small-scale and traditional populations, the accrual of wealth and status carried positive fitness benefits (Kaplan, 1996; Sear, Lawson, Kaplan, & Shenk, 2016; von Rueden, 2014; von Rueden et al., 2011). However, the positive effects of wealth and status on fertility are far from universal, and numerous studies have also shown negative or null associations, especially among market-integrated populations (Retherford, 1986; Vining, 1986).

Within contemporary Western populations, wealthier, higher status men tend to have lower fertility (Kaplan et al., 2002; Lam, 1986; Pérusse, 1993). In low and middle-income countries where populations are at different stages of this transition, women in wealthier households have fewer children on average (Hruschka & Burger, 2016; Hruschka, Sear, Hackman, & Drake, 2019; Lutz & KC, 2011; Myrskylä, Kohler, & Billari, 2009; Pérusse, 1993). Over the course of the fertility transition, wealthier families also reduce their fertility earlier and more dramatically than the rest of the population (Borgerhoff Mulder, 1998; Livi-Bacci, 1986; Skirbekk, 2008). Finally, studies using historical samples have identified a switch in the relationship between socioeconomic status and fertility, whereby high-status individuals move to low-fertility strategies while low-status individuals move to having relatively higher fertility (Skirbekk, 2008). Scholars have argued that these findings show that the links between fitness and the accumulation of wealth and status have been disrupted or severed in contemporary populations (Colleran, Jasienska, Nenko, Galbarczyk, & Mace, 2015; Pérusse, 1993). Indeed, studies of low-fertility regimes have shown that increases in wealth and status at the expense of fertility have never resulted in a long-term fitness payoff (Goodman, Koupil, & Lawson, 2012; Kaplan, Lancaster, Johnson, & Bock, 1995).

The current paper addresses the puzzling and inconsistent associations between socioeconomic status and fertility by exploring the possibility that measures of socioeconomic status (e.g. income, wealth & education) confound two conceptually distinct factors – differential economic capacity and differential market opportunities – that should have opposing effects on fertility. We first review recent studies and theoretical explanations of the wealth–fertility association. We focus on investment models, common in economics and evolutionary social sciences, which argue that market economies drive down fertility as parents focus on generating access to novel forms of social and economic opportunities for their children (Shenk, 2009). Next, we argue that commonly used measures of wealth tend to conflate economic capacity with market opportunities, making it difficult to test theories about the independent effects of economic capacity and market opportunities. Finally, we attempt to disentangle the effects of market opportunities and economic capacity on fertility using multiple forms of wealth to parse out the effects of economic capacity from those of market opportunities.

### Market economies and changes in quality–quantity trade-offs

The demographic transition and associated changes in the wealth and fertility relationship have been discussed in great detail in evolutionary social sciences (Borgerhoff Mulder, 1998; Irons, 1983; Mace, 1996; Sear et al., 2016; Vining, 1986). The causes of the fertility decline and changing relationships between socioeconomic status and fertility have been attributed to increasing costs and benefits of status competition (Boone & Kessler, 1999; Borgerhoff Mulder, 1998; Hill & Reeve, 2005; Low, Simon, & Anderson, 2002; Mace, 2008), the increasing costs and benefits of parental investments in novel market economies (Becker, Murphy, & Tamura, 1990; Kaplan, 1996), women's education (Low et al., 2002), changing payoffs to human capital investments (Kaplan, Hill, Lancaster, & Hurtado, 2000), the breakdown of kinship networks (Newson, Postmes, Lea, & Webley, 2005; Turke, 1989), cultural evolution (Boyd & Richerson, 1985; Richerson & Boyd, 2005), or the costs and benefits of fertility reduction as a social mobility strategy in a stratified society (Rogers, 1990).

Perhaps the most prominent evolutionary and economic explanations have been those that focus on how market economies shape the quality–quantity trade-off that parents face. These models focus on life-history trade-offs where parents balance investment in producing offspring with investment in the quality of existing offspring to promote survival and future success (Lawson, Alvergne, & Gibson, 2012; Lawson & Mace, 2011; Lawson & Mulder, 2016).

**Figure 1.**
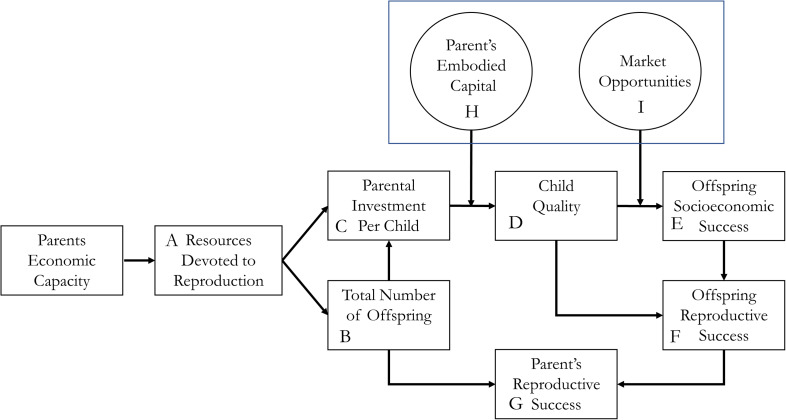
Theoretical model of the quality quantity trade-off.

Figure 1 outlines the key points of the quality–quantity models. Resources that are devoted to reproduction (A) are split between the total number of offspring (B) and the investment in each offspring (C) in order to maximise a parent's reproductive success (G). Kaplan and colleagues developed an extensive theory of human reproduction that specified many of the links in the full model (Kaplan, 1996; Kaplan et al., 2002; Snopkowski & Kaplan, 2014). According to their model, among hunter–gatherers, fertility is coordinated through systems of behavioural and physiological responses, with psychological adaptations which evolved to track the links between parental investment and the reproductive success of their offspring. The skill-dependent foraging niche of humans required extended parental support, which placed constraints on the fitness returns to both parental investments in existing offspring, and the production of additional offspring.

A key premise in this model is that navigating trade-offs in order to maximise reproductive success (G) is a complex task. Thus, parents try to optimise some combination of proximate currencies like total offspring (B) and offspring socioeconomic success (E).

To do this, parents will pay attention to how the investments in child quality (C) translate to child quality (D) and how child quality translates into socioeconomic success (E). These links are indicated by dashed lines in Figure 1. The model also proposes that parents use offspring quality (D) and offspring socioeconomic (E) as proxies for the more distal variables of offspring reproductive fitness, and do not pay particular attention to the downstream links between D and F, or E and F. Thus, when these links become broken (as they are in contemporary societies), the argument is that these do not hold strong sway over decision-making (Goodman et al., 2012).

According to this model, increasing socioeconomic payoffs to investment in child quality can tip the balance of quality–quantity trade-offs, leading parents to reduce fertility in favour of investing in fewer high-quality children. The model here outlines two ways that returns on investment can be increased. The first is increasing the efficiency with which parental investments (C) translate into child quality (D), for example investments in education leading to a more educated and skilled child. The second is how well a person can use their education and skills to unlock opportunities for increased income or socioeconomic success. The embodied capital model touches on both of these. First, it proposes that parents with experience in the education system are better equipped to help their children navigate the educational system, and their investments in education can more efficiently lead to an educated child (Kaplan, 1996; Kaplan et al., 2002). This can increase the efficiency of translating investments to educational achievement, and lead parents with more embodied capital (H) to redirect investments to offspring quality at the expense of offspring quantity. Second, and critical to the current study, the model also considers how changing market opportunities can increase the socioeconomic returns to education (e.g. the efficiency by which D translates to E). Competitive wage-labour economies increase opportunities to translate current child quality (i.e. education) into future offspring socioeconomic success. Furthermore, changing balances of supply and demand for skilled labour can affect returns on investment in child quality (Kaplan & Lancaster, 2000).

While broad social changes can shape overall quality–quantity trade-offs, not all children in a population will have the same opportunities to translate education (or other investments in quality) into greater later life success. For example, changing economic conditions may affect women differentially because of lower expected socioeconomic gains from education and differences in available employment opportunities (Snopkowski & Kaplan, 2014). A family's social connections may provide more opportunities for translating a child's education into income-producing jobs (Coleman, 1988; Matthews, Pendakur, & Young, 2009; Portes & Landolt, 1998). The wage returns to schooling may be lower for ethnic groups that suffer from exclusion from certain sectors of the labour market (Patrinos, 2000; Patrinos & Psacharopoulos, 1997). Even physical proximity to market opportunities may shape returns to investment (McAllister, Gurven, Kaplan, & Stieglitz, 2012). Market opportunities are often more concentrated in urban centres, providing more opportunities to translate education into income-producing employment (Mattison & Neill, 2013; Neill, 2010). Additionally, a family's experience with labour markets and market economies may provide valuable skills and connections necessary to translate education into future income and socioeconomic status. Even in the presence of educational opportunities, family livelihoods may shape the expected returns to, and willingness to invest in, offspring education (Hedges, Borgerhoff Mulder, James, & Lawson, 2016).

In each of these cases, individual and household variation in *market opportunities* – or the suite of options to translate education into future socioeconomic status – may substantially shape the returns on investment in child quality and quality–quantity trade-offs. Analyses of populations without accounting for this variation in accessing market opportunities may obscure how parents make decisions about the trade-offs (Stulp, Sear, & Barrett, 2016). For example, in heterogeneous populations, where different groups of people face unique suites of employment opportunities, investment options, or different costs of raising children, groups with greater market opportunities might follow decision rules that lead to smaller families overall compared with groups with fewer market opportunities (Mace, 1998; Stulp & Barrett, 2016). In those cases where family status (however measured) is associated with greater market opportunities for their children, this can lead to a negative correlation between family status and fertility at the population level. However, within those high- and low-status groups we may see those with greater economic capacity still having higher relative fertility (Mace, 1998). In an early empirical example, one study among highly educated women in Britain's top universities found wealth was positively associated with reproductive success (Hubback, 1957). Here, similarities in education can be assumed to reflect similarities in market opportunities. In another example, across subgroups in urban and rural Mongolia, researchers found a negative association between resources and fertility; however, within each group fertility correlated positively with measures of material resources (Alvergne & Lummaa, 2014). More recently, Colleran found that within communities in rural Poland, associations of non-farming and farming wealth with fertility were generally positive after controlling for market integration and education (Colleran et al., 2015).

Notably, this theoretical model also illustrates the various ways that the concept of status can figure in fertility decisions, and the difficulties of clearly differentiating wealth and status. One approach distinguishes wealth defined as resources from status defined as access to resources (Colleran et al., 2015; Colleran & Snopkowski, 2018). According to this definition, a number of key aspects of the model fall under the concept of status. Parents’ education (under embodied capital H) provides access to cultural capital that can increase the efficiency of translating parental investments in child education into child quality. Parents’ and children's access to market opportunities (I) and children's socioeconomic success (E) are also forms of status, defined again as access to resources. Even parent's economic capacity – which is an intuitively straightforward measure of material wealth – is an indicator of their ability to access resources through economic exchange, and thus a form of status as well.

Another approach defines status as a positional good where the value is defined relative to the amount held by others in a relevant community, whereas wealth is defined as an absolute good whose value is fixed (Shenk, Hooper, & Kaplan, 2016). However, it can be difficult to disentangle what resources are absolute and what resources are relative in a given context. For example, parental embodied capital (H), offspring quality (D) and socioeconomic success (E) can be defined as relational goods, where the value depends on how much is present in a relevant community. Even parental economic capacity can be a relative concept. For example, the economic value of money can change depending on how much others in the monetary system control. More generally, the status–wealth distinction can be difficult to parse empirically, as resources and access to resources can by proxied by the same measures, and absolute and relational goods are often not clearly demarcated in the real world. The multidimensional and entangled nature of both wealth and status suggests that simple status–wealth distinctions may not be sufficient to understand the effects of resources on fertility. Rather, models may need to carefully specify how different kinds of resources, access and opportunities shape fertility-related decisions and how a single measure may capture several kinds of resources, access and opportunities that are relevant to fertility in different ways.

### Assessing support for the model is difficult due to data and measurement issues

Researchers have argued that data limitations have inhibited clear tests of the model, particularly in comparative contexts (Colleran et al., 2015; Hopcroft, 2006; Stulp, Sear, Schaffnit, Mills, & Barrett, 2016). A key component of this critique is disentangling key concepts, such as economic capacity and market opportunity, from standard measures of socioeconomic status. Disentangling economic capacity and market opportunities is important because they are argued to have distinct, interactive or even opposing effects on fertility (Colleran et al., 2015; Shenk et al., 2016). However, historical and cross-sectional datasets often use measures that conflate market opportunities with economic capacity. Different measures of socioeconomic status may differentially confound economic status and market opportunities available to their children. For example, parental education may more strongly reflect market opportunities than economic capacity. Consistent with this expectation, parental education is robustly associated with investing more in each of fewer children (Skirbekk, 2008).

Even something as apparently simple as parental wealth may reflect both parental economic capacity and market opportunities available to their children. For example, asset-based wealth measures commonly used in large demographic datasets most commonly reflect the kinds of assets that can be accumulated with cash and engagement in market economies (e.g. TVs, cell phones, concrete walls, tin roofs; Bingenheimer, 2007; Hruschka, Hadley, & Hackman, 2017; Rutstein & Johnson, 2004). As such, these measures reflect the outcome of a history of engagement in specific types of economic production and exposure to distinct suites of opportunities to accumulate different sorts of material assets and manage reproduction. For this reason, these measures of material wealth reflect not only the economic capacity of a household, but also the extent to which households are exposed to, and can capitalise on, market opportunities.

In contrast to a one-dimensional model of material wealth, a multidimensional model of wealth suggests that different asset-based indicators of wealth reflect not only the economic capacity of a household, but also a proxy for engagement in different types of economic production. Engagement in these distinct dimensions may create suites of opportunities and constraints for households that are not captured in one-dimensional approaches to estimating material wealth. These opportunities and constraints are particularly important for understanding the nature of the reversals between wealth and fertility. For example, success in the agricultural economy requires that parents make very different decisions regarding investments in human capital compared with households engaged in the livelihoods grounded in the cash economy. The returns to investments in education and to reduced fertility may vary widely when households are predominantly engaged in professional wage-labour vs. predominantly agricultural production (Hedges et al., 2016).

To address this concern, recent studies have focused on agricultural measures of material wealth and the effects on fertility, particularly in mixed economies where traditional livelihoods exist alongside market opportunities (Colleran et al., 2015; Garenne, 2015). In previous work, Colleran has pointed to the need to include multiple forms of wealth, particularly traditional and market-based, as a means of disentangling economic capacity and engagement in the market economy. Indeed, in her study of farming communities in rural Poland she includes measures of both farming and non-farming wealth, based on ownership of different suites of assets. Furthermore, she used independent indicators of market integration using employment status and occupation categories. The ability to capture more direct measures of ‘traditional’ and ‘modern’ wealth is a strength of using primary data in small-scale or regional studies. However, these studies tell us little about the effects across a broader range of global contexts. To better understand the process of demographic change in the contexts of market integration, the changes in the importance of different types of material wealth and the suites of opportunities that accompany economic development, we need comparative data.

## The current study

**Figure 2.**
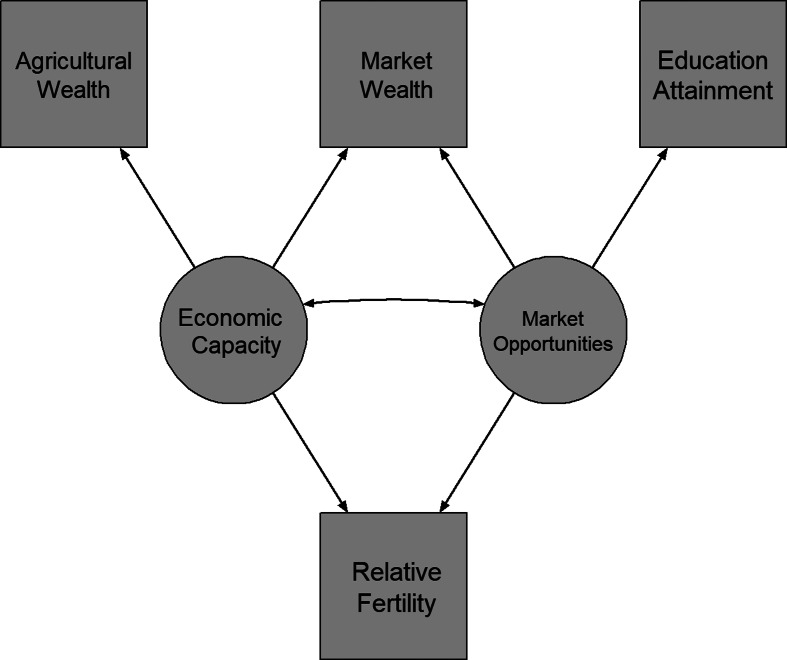
Latent variable model estimating economic capacity and market opportunities.

The current study proposes and tests a method for parsing the effects of economic capacity from market opportunities using multiple measures of wealth. To do so, we treat economic capacity and market opportunities as underlying latent traits, which are partially captured by measures of education and wealth along different dimensions (Figure 2). We assume that both market-based wealth and educational attainment to some extent reflect a history of the market opportunities available to a household. Similarly, we expect market-based wealth and agricultural-based wealth to both reflect the economic capacity of a household. Using multiple empirical measures to estimate economic capacity and market opportunities as latent variables permits a more direct test of the links between fertility and what anthropologists consider wealth and status.

We use this approach on a large database of demographic and health monitoring surveys from low- and middle-income countries worldwide to assess the overall effect of economic capacity and market opportunities on fertility. These surveys have the advantage of containing information about both cash economy market wealth (e.g. TVs, cars) and agricultural wealth (e.g. land and livestock) (Garenne, 2015; Hackman, Hruschka, & Vizireanu, 2020; Hruschka et al., 2017). Using both measures of wealth in a latent variable structural equation model, we estimate how agricultural and market-based wealth reflect economic capacity, and how education and market wealth reflect market opportunities. The modelling approach permits estimation of (a) the economic capacity and market opportunities as independent latent variables and (b) their overall effect on fertility outcomes across a broad range of populations currently undergoing transitions to low fertility.

## Data and methods

### Data and general approach

We analyse data from 151 Demographic and Health surveys (DHS, 1989–2016). The DHS are nationally representative surveys that collect data in low- and middle-income countries. Most DHS surveys contain detailed information on reproduction for women aged 15–49 as well as household economic status. We use available data from 64 countries with reliable estimates of market and agricultural dimensions of wealth (Hackman et al., 2020) and data on completed fertility for women aged 40–50 (562,324 women, across 111,724 sampling clusters; Figure S1 and Table S1 in the Supporting Information). The surveys reflect populations in Europe and Central Asia (*n =* 11), South Asia and Oceania (*n =* 10), sub-Saharan Africa (*n =* 92), the Middle East and North Africa (*n =* 5), Southeast Asia (*n =* 16) and Latin America and the Caribbean (*n =* 17). Given that the nature and meaning of wealth may vary across contexts with different suites of social and economic opportunities for pursuing different livelihoods, we also conduct analyses stratified by urban vs. rural residence. Sample sizes varied across the 151 surveys, ranging from a low of *n* = 322 for Malawi 2012 to a high of *n* = 149,295 for India 2015.

### Measures

#### Wealth indices

We follow the procedure outlined in prior publications to estimate orthogonal measures of market-based and agricultural wealth using multiple correspondence analysis (MCA) (Hackman et al., 2020; Hruschka et al., 2017). Household-level wealth dimensions were computed for each survey independently, as the MCA provides estimates of relative ranking of households based on similar asset ownerships within the population, but is not strictly comparable across surveys. Variables used to estimate the dimensions included source of drinking water, electricity, wall, roof and floor construction material, sanitation, cooking fuels and material asset ownership like bikes, radios, televisions and cell phones. Household holdings of land and livestock (e.g. cattle, cows, sheep, chickens, horses, goats and country-specific animals) were also included. The MCA produced two wealth dimensions that had clear interpretations of reflecting either the market economy or the agricultural sector when validated against agricultural indicators (e.g. livestock, and land) and market-oriented goods (e.g. TVs, cars) (Hackman et al., 2020). These dimensions are scaled to have a mean standard deviation (SD) of 1 within surveys. We excluded seven (of 158 total) surveys that had a low internal reliability of the agricultural wealth dimension (Cronbach's *α*< 0.6, see Figure S8 for distribution). A rule-of-thumb threshold for acceptable reliability is typically 0.7, as lower *α* scores suggest that the wealth dimension is not sufficiently reliable to warrant a clear interpretation. The MCA produces a ranking of households along each dimension for all households included in that survey. In other words, the indices produce a survey-level relative ranking of households along each of two wealth dimensions. For example, a household that owns neither land or livestock would be ranked relatively low on the agricultural wealth dimension. Notably, in the low- and middle-income countries considered here, many urban households own agricultural assets, and many rural households own no agricultural assets. Thus, one frequently finds urban households having higher agricultural wealth dimensions than rural households.

#### Education

We use number of years of education which ranged from 0 to 15 across all surveys (Snopkowski, Towner, Shenk, & Colleran, 2016).

#### Total fertility

We used total children ever born for all women aged 40–50 years old. Survey average completed family size ranged from a low of 1.8 in Ukraine 2007 and 2.3 in Moldova 2005 to a high of 7.6 in Niger 2012 and 7.5 in Chad 2015 (Table S1). Across all surveys the mean total fertility was 5.2 (SD = 1.3, range between 5 and 95% percentiles 2.9–7.1). The distributions of survey-level fertility and fertility variance are reported in Figure S7.

#### Centering on sampling cluster

To assess the local, relative effects of both measures of wealth, we centre each household's measures of wealth, fertility and education relative to the local cluster mean. The sampling cluster is the lowest level of geographic scale in the DHS sampling strategy. Clusters reflect sampling units that represent about 20–50 households that are in relatively close proximity to each other (mean 43.9, SD = 91.7). Some 95% of the primary sampling units (PSUs) have between 1 and 80 individuals represented in the dataset, despite the range realistically going as high as 776. To facilitate centring, we exclude 317 sampling clusters with *N* = 1.

This centring permits estimation of a household's economic position relative to the community mean rather than the country (Colleran & Snopkowski, 2018). This is the level at which we would expect to observe the strongest effects of resources and opportunities on fertility (Gibson & Lawson, 2011; Nettle & Pollet, 2008). It is precisely in these local contexts where the distributions of opportunities and resources shape what economic pathways are feasible. Furthermore, local social contexts provide the suite of behavioural strategies deemed acceptable behaviour (Bachrach & Morgan, 2013; Colleran et al., 2014; Johnson-Hanks, Bachrach, Morgan, & Kohler, 2011). Centring all measures at the cluster level permits estimation of the relative effects of wealth and education on fertility while holding community-level differences constant.

### Analyses

To test whether market-based wealth measures conflate economic capacity and market opportunities, we employ a latent variable structural regression model (Kline, 2015). In the model, we use market wealth, agricultural wealth, and education to estimate two latent factors – economic capacity and market opportunities – which are then regressed on relative fertility (see Figure 2). This model aims to disentangle the effects of market opportunities from economic capacity in standard asset-based wealth measures. Thus, it provides a first approximation of the relative magnitude of the positive effects of economic capacity on fertility relative to the negative effects of market opportunities.

We estimate a total of four structural equation models (SEM). We first run the model on the full sample, without accounting for population differences. We then stratify this model by urban and rural residence to assess how the effects of economic capacity and market opportunities might vary across broad contexts. Finally, we estimate the full model for each population in the sample using multigroup specification. The multigroup SEM runs an independent model for each survey, producing both a survey-specific estimate and an average estimate of the effect. We examine the sign and magnitude of the regression parameters in the structural model for each population to assess whether economic capacity and market opportunities are associated with relative fertility in the predicted directions. We expect a positive effect of economic capacity on fertility while market opportunities should have a negative effect.

The results of the measurement submodel – the part of the model estimating the latent variables from the measures of wealth and education – are presented as the fully standardised solution (the standardized beta coefficient β) in Table 1. This allows for interpreting the factor loading coefficients on each latent variable as standardised regression coefficients, which permits comparisons within and between models on a similar metric. This standardised solution allows for a clearer interpretation of the factor loadings on the latent variables, whereas, for the structural submodel, the coefficients are presented as the unstandardised coefficients. In contrast to the measurement portion of the model, the unstandardised coefficients have a meaningful interpretation – a standard deviation increase in the latent variable reflects the average increase in number of children relative to the community mean.

**Table 1.** Results of the SEM models

	Full sample		Urban sample		Rural sample		Multigroup sample^a^	
Model summary and fit	Test statistic	*p*-Value	Test statistic	*p*-Value	Test statistic	*p*-Value	Test statistic	*p*-Value
*χ*^2^ test	3295.98	<0.001	856.04	<0.001	3653.10	<0.001	8695.21	<0.001
RMSEA	0.04	1.00	0.03	1.00	0.05	0.16	0.06	0.001
CFI	0.97		0.99		0.93		0.94	
d.f.	4		4		4		604	
Groups	1		1		1		151	
*N*	562,324		209,032		353,292		562,324	
Measurement submodel	Standard *β*	SE	Standard *β*	SE	Standard *β*	SE	Mean standard *β*	Mean SE
*Latent factor loadings*								
Market wealth Economic capacity^a^	0.20	0.002	0.38	0.002	0.09	0.002	0.16	0.047
Agricultural wealth^a^ Economic capacity	0.61	0.001	0.70		0.58		0.61	0.010
								
Market wealth^a^ Market opportunities	0.38	0.002	0.38	0.002	0.34	0.001	0.34	0.039
Education^a^ Market opportunities	0.80	0.001	0.71		0.88		0.77	0.013
*Latent covariance*								
Market opportunities Economic capacity^a^	0.27	0.003	0.38	0.004	0.21	0.003	0.30	0.052
								
Structural regression submodel	*β*	SE	*β*	SE	*β*	SE	Mean *β*	SE
Intercept	0.00	0.00	0.00	0.01	0.00	0.00	0.00	0.10
Economic capacity	0.13	0.01	0.17	0.01	0.17	0.01	0.29	0.14
Market opportunities	−0.53	0.00	−0.76	0.01	−0.39	0.01	−0.70	0.13

a The multigroup specification presents the coefficients averaged across all individual survey models.

For each of the SEM models, we assess model fit using a number of model fit indices. We use the standard *χ*^2^ test to assess model fit; however, this test becomes less reliable with large samples, and very small discrepancies can result in failing this model test (Kenny, 2014). We also report the RMSEA, which is a widely used measure of model fit and is relatively invariant to large increases in sample sizes. The RMSEA has standard thresholds of less than 0.05 for a good fit (Kline, 2015). Finally, we include the comparative fit index (CFI), which compares the model fit with the null model, and has standard threshold cutoffs of greater than 0.95 as indicating good fit (Schreiber, 2006).

In SEM models, estimating a latent variable requires a factor loading to be fixed in order to scale the latent variables (Kenny, 2014). We fix the factor loadings of agricultural wealth as the indicator for economic capacity, and education as the indicator for market opportunities. For the full model and urban–rural stratified models, we fix the factor loadings to 0.5, which scales the latent variable variance to the units of the indicator variable. For example, a 0.5 increase in the Agricultural Wealth Index is associated with a 1 SD increase in economic capacity. However, our multigroup model was sensitive to the fixed values of the indicator variables, and convergence issues occurred when setting values to 0.5. Running a multigroup SEM across 151 different surveys with standard fixed factor loadings can result in convergence issues, particularly with a measurement submodel that has relatively few indicators. Most prominent are Heywood cases, where estimated variances are negative. Fixing both indicator factor loadings to 0.5 produced 32% significant negative variance cases across all 151 studies. Rescaling the latent variables by setting one of the reference indicators (education attainment) to 0.35 rather than the standard 0.5 resulted in only 2% of cases having significant negative variances. Here we report results of the multigroup model with the fixed indicator set to 0.35. A sensitivity analysis shows that changing the fixed indicator values had little effect on estimated model parameters (Figures S4 and S5).

## Results

### Model estimation and model fit

**Figure 3.**
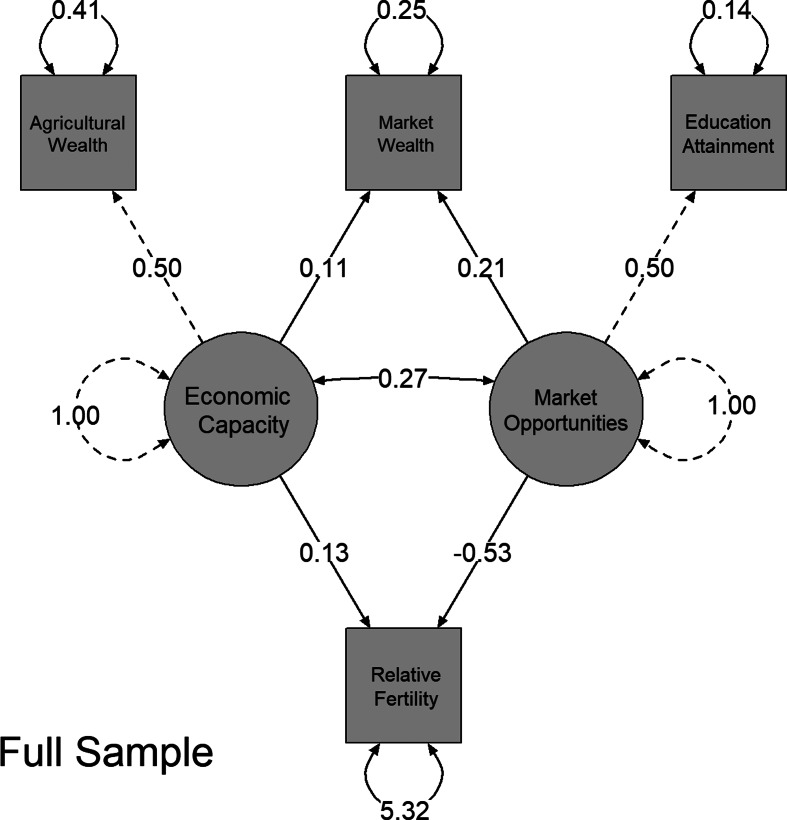
Unstandardised SEM results for the full sample SEM model.

The results of the four SEM models are presented in Table 1 and Figure 3. The *χ*^2^ test was significant, indicating a poor fit for all models; however, this is probably the result of large sample size capturing small differences (Kenny, 2014). Assessment of model fit, using measures less sensitive to sample size, showed acceptable fits for all models. The full model showed good fit across all indices [RMSEA = 0.038, 95% CI = (0.037–0.039), CFI = 0.97]. The urban model similarly showed good fit across all indices [RMSEA = 0.03, 95% CI = (0.030–0.034), CFI = 0.99], while the rural sample model showed acceptable fit [RMSEA = 0.05, 95% CI = (0.049–0.052), CFI = 0.93]. Finally, the aggregate fit indices for the multigroup showed acceptable fit with RMSEA = 0.058, 95% CI = (0.056–0.059) and good fit with CFI = 0.95. Taken together, the four models fit the data well, with the unstratified and urban models showing the greatest fit.

### The measurement submodel: estimating the latent variables

#### Full sample

In the full sample, both agricultural wealth (standard *β* = 0.61, *p* < 0.001) and market-based wealth (standard *β* = 0.20, *p* < 0.001) load positively on the latent variable economic capacity (Table 1). While the MCA produces orthogonal dimensions, when clustering at community-levels these two dimensions show a small positive association (*r* = 0.19, *p* < 0.001). The factor loading for agricultural wealth on economic capacity is three times higher than the factor loading for market wealth on economic capacity. Both education and market wealth had positive and significant loadings on market opportunities (market wealth standard *β* = 0.38, *p* < 0.001 and education standard *β* = 0.80, *p* < 0.001). Finally, economic capacity was positively associated with market opportunities (standard *β* = 0.27, *p* < 0.001).

#### Urban vs. rural sample

The urban and rural stratified results were qualitatively similar to the full unstratified model, with a few exceptions. First, factor loadings of market wealth on economic capacity, were weaker in rural settings (standard *β* = 0.09, *p* < 0.001) than in urban settings (standard *β* = 0.38, *p* < 0.001). Second, the associations between the latent variables, economic capacity and market opportunities were weaker in rural settings (standard *β* = 0.21, *p* < 0.001) than in urban settings (standard *β* = 0.38, *p* < 0.001).

#### Multigroup sample

In the multigroup sample both market wealth (standard *β* = 0.16, *p* < 0.001) and agricultural wealth (standard *β* = 0.61, *p* < 0.001) loaded positively on economic capacity, while market wealth (standard *β* = 0.34, *p* < 0.001) and education (standard *β* = 0.77, *p* < 0.001) loaded positively on market opportunities. The average association between the economic capacity and market opportunities was also positive and significant (standard *β* = 0.30, *p* < 0.001).

### The structural submodel: predicting fertility by economic capacity and market opportunities

In the full sample model, a 1 SD increase in the Economic Capacity latent variable was associated with a small but significant increase in fertility (*β* = 0.13. 95% CI = (0.12–0.14), *p* < 0.001). Similar effects were observed in the urban and rural models [urban *β* = 0.17, 95% CI = (0.15–0.18), *p* < 0.001, rural *β* = 0.17, 95% CI = (0.15–0.18), *p* < 0.001], while the mean effect was slightly larger in the multigroup models (mean *β* = 0.21). These estimates correspond to approximately 0.50–0.85 more children for women in households with 4 SD greater economic capacity.

In contrast, market opportunities showed strong negative effects on fertility in all models. A 4 SD increase in market opportunities was associated with a decline of approximately two children in both the full model [*β* = −0.53, 95% CI = (−0.54 to −0.52), *p* < 0.001] and the multigroup model (mean *β* = −0.60, *p* < 0.001). The effect of market opportunities on fertility was weaker in the rural sample [rural *β* = −0.39, 95% CI = (−0.40 to −0.38), *p* < 0.001] than in the urban sample [urban *β* = −0.76, 95% CI = (−0.77 to −0.74), *p* < 0.001]. The ratio of the effects of both latent variables on fertility shows that the positive effects of economic capacity ranged from one-fifth the size of the negative effect of market opportunities in urban settings, to over one-third the size in rural settings.

#### Multigroup SEM results

**Figure 4.**
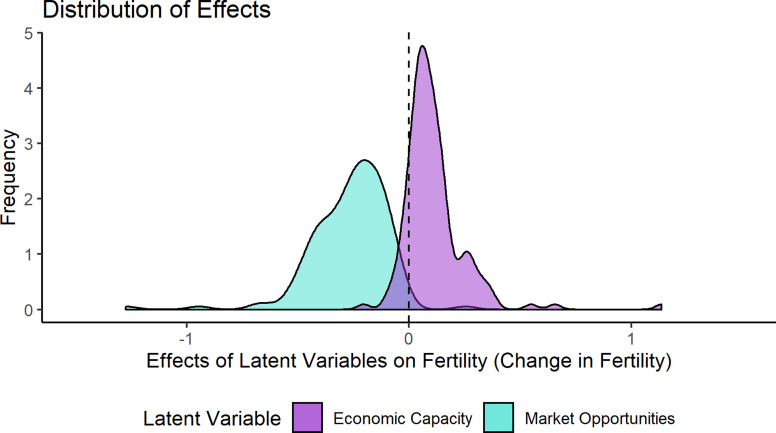
Distribution of effects of latent variables on fertility.

**Figure 5.**
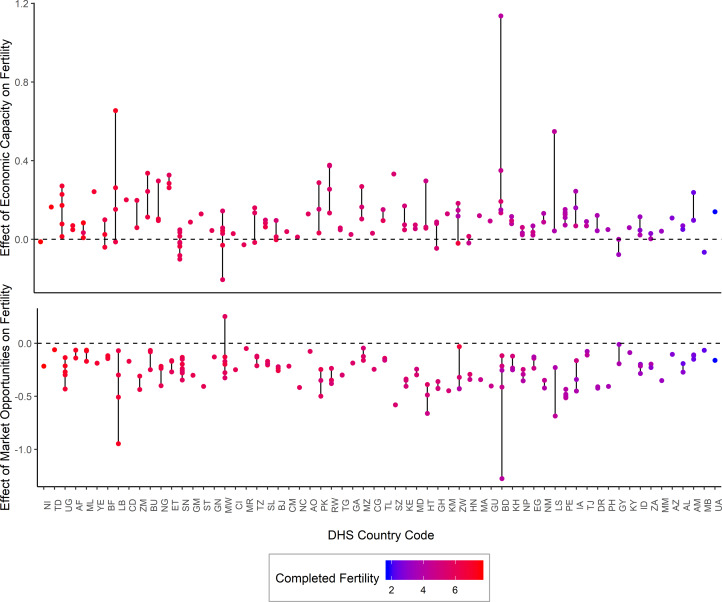
Distribution of effects of latent variables on fertility across survey waves. The top panel shows the effects of economic capacity on fertility for multiple survey waves within a country. The effects were largely positive with within countries across survey waves. The bottom panel shows that the effects of market opportunities were similarly consistent within countries across survey waves. Countries are ordered by average completed fertility across all survey waves (Tables S1 and S7).

The effects of market opportunities and economic capacity on fertility across surveys and countries showed substantial variation (Figures 4 and 5). Overall, market opportunities had a consistent negative effect on fertility across 99.3% of the 151 surveys included in the model. In contrast, economic capacity had an average positive effect, with 87.4% positive. The remaining 12.6% showed null effects of economic capacity (See Figure S3). The effects of market opportunities showed a range of variation across countries with the 5–95% percentile of the distribution between −0.06 and −0.52. The effects of economic capacity similarly showed a range of variation, with the 5–95% range of the distribution from −0.03 to 0.33.

## Discussion

These results suggest a new approach to assessing the associations between measured wealth and fertility found in populations undergoing fertility transitions. Rather than capturing fertility differences between households with different levels of economic resources, these associations are capturing households that vary not just in terms of their economic capacity, but also in their access to market opportunities needed to turn high-cost investments into greater socioeconomic success. Furthermore, when accounting for variation in market opportunities, households with greater economic capacity still tend to have relatively higher fertility than households with less economic capacity. This suggests that many of the puzzling associations between wealth and fertility may reflect the inability of current approaches to properly disentangle market opportunities and economic capacity. Indeed, the majority of economic and evolutionary theories of low fertility point to the influence of modern competitive wage labour economies as the primary driver of fertility declines. However, as market economies develop and take hold in a population, households vary in their ability to access and take advantage of novel economic opportunities offered by expanding markets. Households with greater opportunities to turn costly investments in education into greater socioeconomic success are more likely to engage in a low-fertility, high-investment strategy.

This exercise in disentangling economic capacity and market opportunities permits a more direct test of the importance of theoretically relevant constructs, such as market opportunities and economic capacity. For standard asset-based wealth measures used in demographic and health surveys, the bias towards market-oriented goods and services means that the measure tracks not only household economic capacity but also serves as a proxy for market opportunities. And if the effects of market opportunities are much larger than the effects of economic capacity (as observed here), then commonly used asset-based measures may tell us comparatively little about economic capacity. When these wealth measures are treated uncritically as simply reflecting economic capacity, they can limit our ability to draw inferences about how economic resources shape reproductive outcomes.

Returning to the theoretical model, the positive association between economic capacity and fertility tells us little about why the links between offspring socioeconomic success (E) and offspring reproductive success (F) are broken or breaking in contemporary societies. In the past offspring quality and socioeconomic status may have served as reliable proxies for the ultimate aim of reproductive success (G). However, in contemporary societies, such proxy relationships can become broken, leading to investments in wealth and status accumulation that do not have associated fitness payoffs (Goodman et al., 2012). Rather than arguing that parental investments are adaptive in these contexts, the model proposes that parents are paying attention to more proximate cues, and may be wholly unaware of the downstream links.

The results also open new questions about how fertility responds to economic development over the course of the fertility transition. For example, what accounts for the considerable cross-national variation in the effects of economic capacity and market opportunities? Potential factors shaping this variation include cultural history, ethnic and economic heterogeneity, the diversity of agricultural livelihoods and their opportunity costs associated with engaging in wage-labour production. Additionally, our approach can be extended to refine operationalisation of other key concepts in theoretical models of fertility. For example, education is often treated as a proxy for status in evolutionary demography (Colleran & Snopkowski, 2018), although recently the effects of education on fertility have been considered multidimensional, capturing more than simply access to resources (Martin & Juarez, 1995), such as exposure to modern low-fertility norms (Colleran et al., 2014), knowledge and ability to control reproduction (Cleland & Kaufmann, 2002; Jayne & Jejeebhoy, 1997; Peña, Wall, & Persson, 2000). Indeed, education seems to have a robust negative effect on fertility although the mechanisms appear to be context specific (Snopkowski et al., 2016). Latent variable analyses may be one means of disentangling these multidimensional effects.

There are a number of important limitations and caveats to interpreting these structural regression models. Primarily, there is a need for additional indicators for the latent variables in order to fully assess the constructs in the measurement model. Our ability to estimate the latent variables rests on the covariance structures in the indicator variables. Here that is the covariance between market-based wealth, and agricultural wealth and education independently. Using only two indicator variables for each latent construct resulted in a relatively unstable model. For example, the SEM model fared worse in rural settings, with relatively poorer scores on model fit indices than in urban settings. One reason for this could be that the low covariance between the indicator variables in rural settings results in poor measurement. The lower covariance between the indicator variables could reflect greater variation in the nature and meaning of the wealth measures, or it could reflect contexts where success along one dimension is not easily transferred to success along another. Without a significant amount of shared variance, it is difficult to estimate reliable latent variables. Another possibility is that the two wealth measures are truly orthogonal and reflect that in many contexts success along one dimension carries steep trade-offs with success along another dimension. To assess these two possibilities, we need more empirical indicators of market opportunities and economic capacity. Two important candidates to include in future work are occupation types and employment status of individuals and heads-of-households. Similar to stocks of assets, these directly reflect a household's engagement with wage-labour employment and also proxy a household's access to competitive labour economies. While the DHS surveys do collect data on occupation and employment, the types of occupations available and their meaning across contexts can vary significantly. Alternatively, future research could use a subset of the underlying asset variables to create the latent variables, rather than the dimension scores produced from the MCA. However, the creation of the MCA dimensions and the process of anchoring the dimensions facilitate a clear interpretation (Hackman et al., 2020; Hruschka et al., 2017). Estimating the latent variables using the raw assets would require an independent method of validating the interpretation of the latent variables. Finally, we would ideally model urban and rural contexts separately within a given survey (see Figure S10). However, the small number of observations in these highly stratified samples led to convergence issues for a substantial portion of samples (30%). Future work on larger samples should permit a clearer understanding of how the effects of wealth on fertility vary across rural contexts in different countries.

Another potential concern is that the positive effects of economic capacity on fertility are largely driven by urban households who are highly integrated into the market economy and also have low agricultural wealth and low fertility. These households might also have low scores on economic capacity owing to their having little or no agricultural wealth, which would create an artificial positive association between economic capacity and fertility. This is an unlikely explanation for several reasons. First, the positive associations of economic capacity and fertility are observed in both rural and urban samples. Second, the correlation between market-based wealth and agricultural wealth is positive in urban settings (not negative) and the covariance between the latent variables economic capacity and market opportunities is positive (Table 1). This shows that urbanites who are poor on agricultural wealth are often poor on market-based wealth as well and that both contribute to low economic capacity and low market opportunities. Additionally, we conducted a sensitivity analyses on the urban subsample, excluding households who were in the bottom 25% of agricultural wealth and the top 25% of market-based wealth. The results were qualitatively similar to those presented in Table 1 (Table S8).

Our results highlight the need to estimate multiple, varying forms of wealth and status in order to disentangle the effects of market opportunities from economic capacity. While anthropologists have focused on estimating multiple forms of wealth and status in community studies, this is difficult in the large secondary datasets often used in evolutionary demography. Our method draws on recent changes to demographic and health monitoring surveys where data on livestock, land and other agricultural assets are used to estimate measures of wealth that track economic capacity independent of market opportunities. Using multiple measures of wealth, we were able to estimate the independent effects of economic capacity and market opportunities on relative fertility. We showed that across a broad range of contexts, economic capacity typically has a positive association with fertility. However, this positive association appears to be masked by a strong, negative association between fertility and a household's engagement in the market economy. Accounting for household-level variation in market opportunities and relying on multiple measures of household economic capacity are key steps to testing and refining theories about how households make reproductive and parental investment decisions both within and between populations.

## Supplementary material

For supplementary material accompanying this paper visit http://dx.doi.org/10.1017/ehs.2020.62. click here to view supplementary material

## Research transparency and reproducibility

The data used for this study are publicly available from the DHS Program (https://www.dhsprogram.com/).
