# Supplementary material for: Disentangling wealth effects on fertility in 64 low- and middle-income countries
Source: Evol Hum Sci. 2020 Nov 23;2:e58. doi: 10.1017/ehs.2020.62 (PMC10427476; doi:10.1017/ehs.2020.62)
Supplement: Supplementary file 1 [file S2513843X20000626sup001.docx]

Supplemental Materials

1. List of DHS Surveys used
2. PSU size distribution
3. Bivariate Correlations of primary variables
4. Distribution of Multi-Group Coefficients
5. Sensitivity Analyses for Multi-Group SEM with alternative fixed indicator values
6. Unstandardized model outputs for full model and stratified urban and rural models.
7. Model effects by survey-level fertility
8. Model outputs for just the most recent survey waves
9. Distribution of fertility and fertility variance across surveys
10. MCA description and Cronbach’s alpha for Agricultural Wealth Dimension
11. Regression Effects of Latent Variables on Fertility by Survey
12. Within country variation in regression effects across survey waves
13. Stratifying models by urban-rural within survey waves
14. Urban sensitivity analysis excluding poor agricultural wealth households

**LIST OF COUNTRIES AND DHS SURVEYS USED**


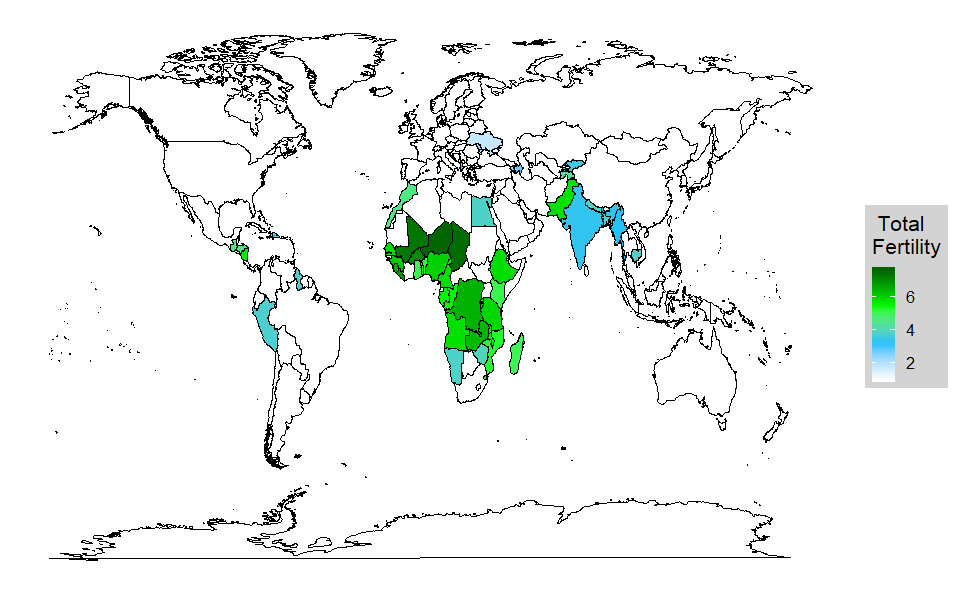


**SM Figure 1. Countries included in the analyses.**

**SM Table 1. List of DHS Surveys**

| DHS Country Code | Survey Year | Sample Size | Mean Cluster Size | Min Cluster Size | Max Cluster Size | Mean Fertility | Std Dev Fertility | Mean Education | Std Dev Education |
| --- | --- | --- | --- | --- | --- | --- | --- | --- | --- |
| Afghanistan | 2010 | 5853 | 71.3 | 30 | 127 | 7.1 | 2.9 | 3.1 | 1.7 |
| Afghanistan | 2015 | 6317 | 32.0 | 12 | 60 | 7.1 | 2.7 | 3.6 | 1.9 |
| Albania | 2008 | 2299 | 18.1 | 5 | 39 | 2.9 | 1.5 | 5.8 | 2.2 |
| Albania | 2017 | 3403 | 23.5 | 4 | 43 | 2.4 | 1.1 | 5.9 | 2.2 |
| Armenia | 2005 | 2024 | 23.1 | 8 | 44 | 2.6 | 1.2 | 3.9 | 1.4 |
| Armenia | 2010 | 1634 | 21.0 | 6 | 40 | 2.4 | 1.1 | 6.4 | 3.4 |
| Armenia | 2016 | 1496 | 21.5 | 5 | 37 | 2.3 | 1.1 | 6.6 | 3.3 |
| Angola | 2015 | 2100 | 24.5 | 5 | 48 | 5.8 | 2.8 | 3.4 | 1.7 |
| Azerbaijan | 2006 | 2327 | 27.8 | 11 | 40 | 2.8 | 1.6 | 6.2 | 1.8 |
| Bangladesh | 1999 | 1988 | 33.0 | 9 | 79 | 5.4 | 2.5 | 3.2 | 1.6 |
| Bangladesh | 2004 | 2260 | 32.1 | 17 | 43 | 5.1 | 2.4 | 2.9 | 1.8 |
| Bangladesh | 2007 | 2282 | 31.0 | 18 | 45 | 4.6 | 2.3 | 3.1 | 1.6 |
| Bangladesh | 2011 | 4005 | 30.4 | 13 | 42 | 4.1 | 2.1 | 3.2 | 1.6 |
| Bangladesh | 2014 | 3984 | 30.6 | 2 | 44 | 3.9 | 1.9 | 3.3 | 1.5 |
| Burkina Faso | 2010 | 2986 | 31.7 | 15 | 67 | 6.8 | 2.4 | 4.0 | 1.6 |
| Burkina Faso | 2014 | 1307 | 34.0 | 16 | 53 | 7.0 | 2.6 | 4.2 | 1.7 |
| Burkina Faso | 2018 | 1217 | 32.6 | 18 | 59 | 6.4 | 2.3 | 3.8 | 1.7 |
| Benin | 2006 | 3049 | 27.0 | 5 | 67 | 6.4 | 2.6 | 3.7 | 1.8 |
| Benin | 2012 | 2805 | 23.7 | 7 | 42 | 5.2 | 2.6 | 3.9 | 1.7 |
| Benin | 2017 | 2606 | 31.3 | 11 | 73 | 5.9 | 2.5 | 3.3 | 1.7 |
| Burundi | 2010 | 1429 | 25.9 | 14 | 52 | 6.5 | 2.7 | 4.5 | 1.8 |
| Burundi | 2012 | 727 | 26.4 | 15 | 48 | 6.4 | 2.7 | 3.9 | 1.8 |
| Burundi | 2016 | 2744 | 31.9 | 19 | 60 | 6.2 | 2.7 | 5.0 | 1.5 |
| Congo Democratic Republic | 2013 | 2918 | 36.7 | 12 | 77 | 6.5 | 3.0 | 3.6 | 1.7 |
| Congo | 2011 | 1927 | 29.6 | 10 | 58 | 5.5 | 2.6 | 3.2 | 1.7 |
| Cote d'Ivoire | 2012 | 1594 | 31.6 | 13 | 59 | 6.0 | 2.9 | 4.3 | 1.7 |
| Cameroon | 2011 | 2461 | 28.8 | 8 | 53 | 5.8 | 3.0 | 4.1 | 1.8 |
| Dominican Republic | 2007 | 6089 | 20.1 | 2 | 39 | 3.8 | 2.2 | 3.9 | 2.2 |
| Dominican Republic | 2013 | 2401 | 18.9 | 2 | 33 | 3.5 | 2.0 | 4.1 | 2.2 |
| Egypt | 2005 | 5453 | 32.6 | 6 | 101 | 4.7 | 2.5 | 4.7 | 1.7 |
| Egypt | 2008 | 4694 | 15.3 | 2 | 38 | 4.4 | 2.3 | 4.9 | 1.8 |
| Egypt | 2014 | 5649 | 30.4 | 2 | 130 | 3.9 | 1.9 | 5.0 | 1.7 |
| Ethiopia | 2005 | 2261 | 27.5 | 6 | 63 | 6.4 | 3.0 | 3.7 | 1.7 |
| Ethiopia | 2011 | 2415 | 28.8 | 5 | 59 | 6.5 | 3.0 | 3.7 | 2.1 |
| Ethiopia | 2016 | 2279 | 26.0 | 7 | 60 | 5.9 | 3.0 | 5.8 | 3.5 |
| Gabon | 2012 | 1611 | 27.4 | 2 | 62 | 5.5 | 3.1 | 3.6 | 1.8 |
| Ghana | 2008 | 920 | 13.0 | 5 | 24 | 5.3 | 2.5 | 3.8 | 1.6 |
| Ghana | 2014 | 1902 | 23.4 | 5 | 45 | 5.1 | 2.4 | 3.6 | 1.4 |
| Ghana | 2016 | 988 | 27.2 | 6 | 49 | 5.1 | 2.5 | 3.3 | 1.4 |
| Gambia | 2013 | 1336 | 42.5 | 14 | 106 | 6.1 | 2.7 | 4.1 | 1.7 |
| Guinea | 2012 | 1622 | 31.9 | 13 | 58 | 6.0 | 2.6 | 3.6 | 1.8 |
| Guatemala | 2015 | 4662 | 31.3 | 7 | 52 | 4.6 | 2.8 | 3.7 | 1.9 |
| Guyana | 2005 | 587 | 21.9 | 7 | 41 | 3.4 | 2.2 | 3.7 | 1.6 |
| Guyana | 2009 | 1189 | 16.8 | 2 | 31 | 3.8 | 2.4 | 3.8 | 1.6 |
| Honduras | 2006 | 3496 | 20.2 | 4 | 39 | 5.5 | 3.0 | 3.8 | 2.0 |
| Honduras | 2012 | 4006 | 20.8 | 2 | 35 | 4.7 | 2.7 | 4.1 | 1.9 |
| Haiti | 2006 | 1924 | 32.5 | 11 | 58 | 5.6 | 3.0 | 3.2 | 1.8 |
| Haiti | 2012 | 2438 | 33.4 | 11 | 57 | 5.1 | 2.8 | 3.3 | 1.8 |
| Haiti | 2017 | 2525 | 36.1 | 12 | 61 | 4.8 | 2.8 | 3.9 | 1.7 |
| India | 1993 | 18703 | 496.6 | 3 | 776 | 4.8 | 2.4 | 3.2 | 1.7 |
| India | 2006 | 22945 | 36.9 | 3 | 98 | 3.8 | 2.1 | 3.8 | 1.6 |
| India | 2015 | 149295 | 25.7 | 3 | 57 | 3.4 | 1.9 | 3.9 | 1.6 |
| Indonesia | 2007 | 9440 | 20.2 | 2 | 34 | 3.8 | 2.2 | 4.4 | 1.8 |
| Indonesia | 2012 | 10906 | 25.9 | 4 | 48 | 3.4 | 2.0 | 4.5 | 1.7 |
| Indonesia | 2017 | 13275 | 26.0 | 5 | 52 | 3.0 | 1.8 | 4.7 | 1.6 |
| Kenya | 2009 | 1400 | 22.7 | 5 | 44 | 5.5 | 2.8 | 4.9 | 2.1 |
| Kenya | 2014 | 5337 | 20.9 | 3 | 44 | 5.3 | 2.6 | 5.2 | 2.2 |
| Kenya | 2015 | 810 | 23.6 | 8 | 39 | 5.1 | 2.7 | 5.3 | 2.4 |
| Cambodia | 2005 | 3771 | 31.3 | 12 | 55 | 5.1 | 2.8 | 2.8 | 1.4 |
| Cambodia | 2010 | 4285 | 31.4 | 15 | 55 | 4.2 | 2.4 | 3.1 | 1.4 |
| Cambodia | 2014 | 3896 | 29.3 | 8 | 50 | 3.8 | 2.2 | 3.1 | 1.5 |
| Comoros | 2012 | 757 | 22.7 | 7 | 42 | 5.1 | 3.0 | 3.9 | 1.7 |
| Kyrgyz Republic | 2012 | 1825 | 27.1 | 10 | 65 | 3.6 | 1.7 | 4.7 | 1.6 |
| Liberia | 2007 | 1275 | 26.2 | 4 | 63 | 6.4 | 2.9 | 3.6 | 1.8 |
| Liberia | 2011 | 643 | 28.3 | 8 | 53 | 7.2 | 3.0 | 3.4 | 2.0 |
| Liberia | 2013 | 1708 | 30.3 | 9 | 51 | 6.6 | 2.9 | 3.1 | 1.9 |
| Liberia | 2016 | 702 | 30.5 | 11 | 59 | 6.8 | 3.1 | 6.1 | 3.5 |
| Lesotho | 2009 | 1310 | 20.2 | 8 | 34 | 4.3 | 2.3 | 4.7 | 2.0 |
| Lesotho | 2014 | 1072 | 18.0 | 5 | 33 | 3.8 | 2.2 | 4.5 | 2.2 |
| Morocco | 2003 | 3564 | 36.6 | 15 | 73 | 4.6 | 3.0 | 3.7 | 1.9 |
| Moldova | 2005 | 2151 | 20.2 | 5 | 39 | 2.3 | 1.2 | 6.6 | 1.8 |
| Madagascar | 2009 | 3144 | 30.3 | 13 | 53 | 5.4 | 3.2 | 3.2 | 1.6 |
| Madagascar | 2016 | 1711 | 31.3 | 15 | 56 | 5.0 | 2.9 | 3.5 | 1.6 |
| Mali | 2001 | 2191 | 34.8 | 9 | 66 | 7.4 | 3.1 | 3.7 | 1.7 |
| Mali | 2006 | 2535 | 37.6 | 10 | 60 | 7.0 | 3.1 | 3.8 | 1.8 |
| Mali | 2012 | 1564 | 26.2 | 11 | 43 | 5.9 | 2.8 | 3.6 | 1.8 |
| Mali | 2015 | 1154 | 47.6 | 21 | 98 | 7.3 | 3.0 | 3.5 | 1.7 |
| Myanmar | 2016 | 3388 | 30.4 | 11 | 56 | 3.3 | 2.5 | 3.4 | 1.5 |
| Mauritania | 2003 | 842 | 31.0 | 13 | 57 | 6.0 | 3.3 | 1.5 | 0.8 |
| Malawi | 2004 | 1710 | 26.0 | 5 | 54 | 6.5 | 2.8 | 4.3 | 2.3 |
| Malawi | 2010 | 3410 | 28.9 | 8 | 59 | 6.6 | 2.7 | 4.4 | 2.4 |
| Malawi | 2012 | 322 | 21.8 | 12 | 37 | 5.8 | 2.7 | 4.8 | 2.2 |
| Malawi | 2014 | 344 | 21.3 | 9 | 34 | 5.8 | 2.6 | 4.7 | 2.4 |
| Malawi | 2015 | 3583 | 30.0 | 9 | 49 | 5.9 | 2.4 | 4.1 | 2.4 |
| Malawi | 2017 | 487 | 26.4 | 17 | 43 | 5.6 | 2.5 | 4.5 | 2.3 |
| Mozambique | 2011 | 2206 | 24.3 | 8 | 49 | 5.3 | 2.8 | 3.4 | 1.8 |
| Mozambique | 2015 | 1199 | 26.1 | 12 | 39 | 5.6 | 2.8 | 4.3 | 2.7 |
| Mozambique | 2018 | 1040 | 29.0 | 10 | 59 | 5.7 | 2.9 | 3.7 | 1.9 |
| Nicaragua | 1998 | 2192 | 26.6 | 2 | 54 | 5.8 | 3.3 | 3.8 | 1.6 |
| Nigeria | 2008 | 5948 | 40.8 | 3 | 76 | 6.6 | 3.0 | 4.7 | 1.6 |
| Nigeria | 2013 | 7218 | 46.5 | 10 | 84 | 6.3 | 3.0 | 4.8 | 1.5 |
| Nigeria | 2015 | 1182 | 26.8 | 5 | 48 | 6.1 | 3.4 | 4.6 | 1.8 |
| Niger | 2012 | 1744 | 25.7 | 6 | 58 | 7.6 | 2.9 | 3.6 | 1.7 |
| Namibia | 2007 | 1664 | 21.5 | 4 | 47 | 4.5 | 2.5 | 3.7 | 1.8 |
| Namibia | 2013 | 1695 | 20.2 | 3 | 43 | 3.9 | 2.3 | 3.7 | 1.7 |
| Nepal | 2006 | 1964 | 43.9 | 21 | 96 | 4.9 | 2.4 | 0.4 | 1.2 |
| Nepal | 2011 | 2216 | 45.4 | 17 | 76 | 4.3 | 2.2 | 2.9 | 1.6 |
| Nepal | 2016 | 2467 | 35.0 | 8 | 61 | 3.9 | 1.9 | 2.9 | 1.6 |
| Peru | 2006 | 2842 | 26.3 | 5 | 55 | 4.3 | 2.7 | 3.8 | 1.4 |
| Peru | 2008 | 5264 | 25.0 | 2 | 49 | 4.1 | 2.6 | 3.8 | 1.5 |
| Peru | 2009 | 5508 | 23.1 | 6 | 52 | 4.0 | 2.5 | 3.8 | 1.5 |
| Peru | 2010 | 5271 | 22.0 | 2 | 48 | 3.9 | 2.5 | 3.8 | 1.5 |
| Peru | 2011 | 5292 | 21.5 | 2 | 44 | 3.9 | 2.4 | 3.8 | 1.5 |
| Peru | 2012 | 5672 | 18.3 | 3 | 37 | 3.8 | 2.4 | 3.9 | 1.5 |
| Philippines | 2017 | 5776 | 21.8 | 3 | 58 | 3.6 | 2.4 | 10.2 | 3.6 |
| Pakistan | 2006 | 2509 | 11.0 | 3 | 23 | 6.2 | 2.9 | 3.9 | 1.4 |
| Pakistan | 2012 | 3482 | 28.2 | 4 | 54 | 5.8 | 2.8 | 4.0 | 1.4 |
| Pakistan | 2018 | 3383 | 27.7 | 7 | 56 | 5.3 | 2.6 | 3.9 | 1.4 |
| Rwanda | 2010 | 2257 | 28.3 | 16 | 52 | 5.9 | 2.6 | 4.3 | 2.2 |
| Rwanda | 2013 | 839 | 32.7 | 22 | 57 | 5.8 | 2.6 | 4.5 | 2.2 |
| Rwanda | 2015 | 2201 | 28.0 | 14 | 47 | 5.4 | 2.5 | 5.1 | 2.3 |
| Rwanda | 2017 | 914 | 29.9 | 17 | 45 | 5.2 | 2.5 | 5.0 | 2.1 |
| Sierra Leone | 2008 | 1178 | 22.4 | 7 | 50 | 5.4 | 2.7 | 3.6 | 1.8 |
| Sierra Leone | 2013 | 2686 | 41.0 | 9 | 92 | 5.9 | 2.7 | 3.6 | 1.7 |
| Sierra Leone | 2016 | 1109 | 26.5 | 12 | 47 | 6.4 | 2.9 | 3.1 | 1.8 |
| Senegal | 2005 | 2261 | 41.7 | 3 | 75 | 6.5 | 3.0 | 4.1 | 1.6 |
| Senegal | 2006 | 1059 | 48.4 | 16 | 88 | 6.5 | 3.0 | 3.7 | 1.7 |
| Senegal | 2008 | 2915 | 64.9 | 25 | 124 | 6.2 | 3.0 | 3.9 | 1.7 |
| Senegal | 2010 | 2313 | 43.7 | 8 | 92 | 6.1 | 3.0 | 4.2 | 1.7 |
| Senegal | 2012 | 1226 | 46.6 | 15 | 80 | 6.3 | 2.9 | 4.3 | 1.6 |
| Senegal | 2014 | 1267 | 46.1 | 9 | 85 | 6.0 | 2.9 | 4.1 | 1.5 |
| Senegal | 2015 | 1267 | 45.0 | 12 | 97 | 6.0 | 2.9 | 4.0 | 1.6 |
| Senegal | 2016 | 1275 | 45.5 | 11 | 114 | 5.9 | 2.9 | 4.0 | 1.6 |
| Senegal | 2017 | 2614 | 45.6 | 10 | 87 | 5.8 | 2.8 | 4.2 | 1.5 |
| Sao Tome and Principe | 2008 | 506 | 32.2 | 8 | 85 | 6.1 | 2.5 | 3.6 | 1.4 |
| Swaziland | 2006 | 825 | 20.3 | 2 | 36 | 5.3 | 2.9 | 4.0 | 1.9 |
| Chad | 2015 | 2893 | 30.1 | 7 | 54 | 7.5 | 2.7 | 3.5 | 1.7 |
| Togo | 2014 | 1751 | 30.7 | 8 | 51 | 5.6 | 2.6 | 3.7 | 1.7 |
| Togo | 2017 | 812 | 28.3 | 12 | 46 | 5.4 | 2.6 | 3.7 | 1.8 |
| Tajikistan | 2012 | 1932 | 28.9 | 7 | 53 | 4.3 | 2.1 | 5.3 | 1.3 |
| Tajikistan | 2017 | 2176 | 30.5 | 13 | 58 | 3.6 | 1.7 | 5.3 | 1.4 |
| Timor-Leste | 2009 | 2494 | 30.5 | 13 | 56 | 5.9 | 2.9 | 4.9 | 2.4 |
| Timor-Leste | 2016 | 2545 | 28.8 | 8 | 53 | 5.0 | 2.7 | 4.5 | 1.8 |
| Tanzania | 2012 | 1894 | 19.8 | 7 | 43 | 6.1 | 2.9 | 6.0 | 1.8 |
| Tanzania | 2015 | 2382 | 23.4 | 8 | 56 | 5.9 | 2.9 | 5.9 | 1.9 |
| Tanzania | 2017 | 1790 | 23.8 | 8 | 55 | 5.8 | 3.0 | 5.9 | 1.9 |
| Ukraine | 2007 | 2028 | 15.7 | 3 | 35 | 1.8 | 0.9 | 5.0 | 1.5 |
| Uganda | 2006 | 1324 | 24.1 | 8 | 43 | 7.3 | 3.1 | 3.9 | 2.1 |
| Uganda | 2009 | 598 | 25.1 | 11 | 42 | 7.0 | 2.9 | 4.2 | 1.9 |
| Uganda | 2011 | 3058 | 25.2 | 11 | 50 | 7.2 | 3.1 | 4.0 | 2.0 |
| Uganda | 2014 | 733 | 27.2 | 9 | 51 | 7.2 | 2.9 | 3.9 | 2.0 |
| Uganda | 2016 | 2805 | 28.0 | 9 | 53 | 6.8 | 2.9 | 4.0 | 1.9 |
| Yemen | 2013 | 3387 | 35.2 | 6 | 67 | 6.9 | 3.1 | 3.6 | 1.9 |
| South Africa | 1998 | 2221 | 14.7 | 2 | 42 | 3.8 | 2.3 | 3.8 | 2.0 |
| South Africa | 2016 | 1868 | 14.5 | 2 | 41 | 2.9 | 1.7 | 3.8 | 1.6 |
| Zambia | 2007 | 995 | 26.1 | 6 | 56 | 6.5 | 3.0 | 4.5 | 2.1 |
| Zambia | 2013 | 2486 | 24.0 | 8 | 52 | 6.4 | 2.9 | 4.4 | 2.1 |
| Zimbabwe | 1988 | 608 | 31.4 | 3 | 74 | 6.6 | 3.2 | 4.3 | 1.8 |
| Zimbabwe | 2005 | 1338 | 24.2 | 7 | 53 | 5.3 | 2.5 | 4.5 | 2.0 |
| Zimbabwe | 2010 | 1359 | 24.2 | 7 | 38 | 4.5 | 2.3 | 4.3 | 1.9 |
| Zimbabwe | 2015 | 1561 | 25.7 | 8 | 44 | 4.0 | 2.2 | 4.0 | 1.9 |

**SIZE DISTRIBUTION OF PRIMARY SAMPLE UNITS (PSU)**


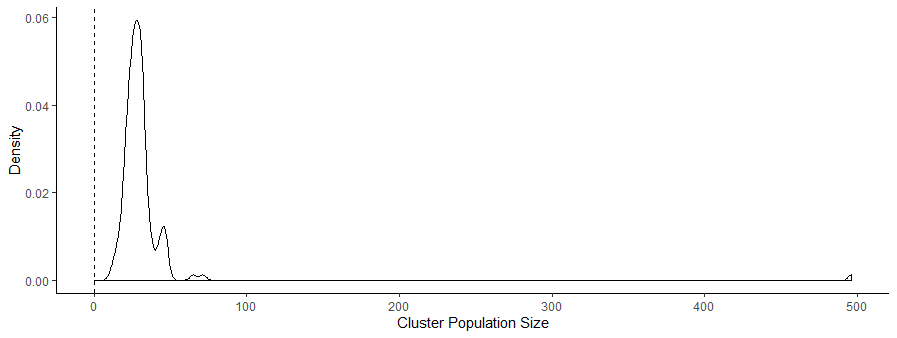


**SM Figure 2. Distribution of mean cluster size across surveys.** The large outlier comes from India 1993 where average cluster size was 496 with a max of 776. Later survey years for India (2006, 20015) had average cluster sizes of 36.9 and 25.7 respectively.

**BIVARIATE CORRELATIONS OF PRIMARY VARIABLES**

| **SM Table 2. Correlations between model variables** | | | | |
| --- | --- | --- | --- | --- |
|  | Completed Fertility | Market Wealth | Agricultural Wealth | Education |
| *Full Sample* |  |  |  |  |
| Completed Fertility | 1.00 |  |  |  |
| Market Wealth | -0.13 | 1.00 |  |  |
| Agricultural Wealth | 0.01 | 0.19 | 1.00 |  |
| Education | -0.16 | 0.36 | 0.14 | 1.00 |
| *Urban* |  |  |  |  |
| Completed Fertility | -1.00 |  |  |  |
| Market Wealth | -0.16 | 1.00 |  |  |
| Agricultural Wealth | -0.04 | 0.36 | 1.00 |  |
| Education | -0.22 | 0.39 | 0.20 | 1.00 |
| *Rural* |  |  |  |  |
| Completed Fertility | -1.00 |  |  |  |
| Market Wealth | -0.12 | 1.00 |  |  |
| Agricultural Wealth | 0.03 | 0.10 | 1.00 |  |
| Education | -0.12 | 0.34 | 0.11 | 1.00 |
| *Study-Average* |  |  |  |  |
| Completed Fertility | -1.00 |  |  |  |
| Market Wealth | -0.12 | 1.00 |  |  |
| Agricultural Wealth | 0.01 | 0.17 | 1.00 |  |
| Education | -0.14 | 0.30 | 0.14 | 1.00 |
|  | | | | |

**DISTIBUTION OF MULT-GROUP STRUCTURAL REGRESSION COEFFICIENTS**


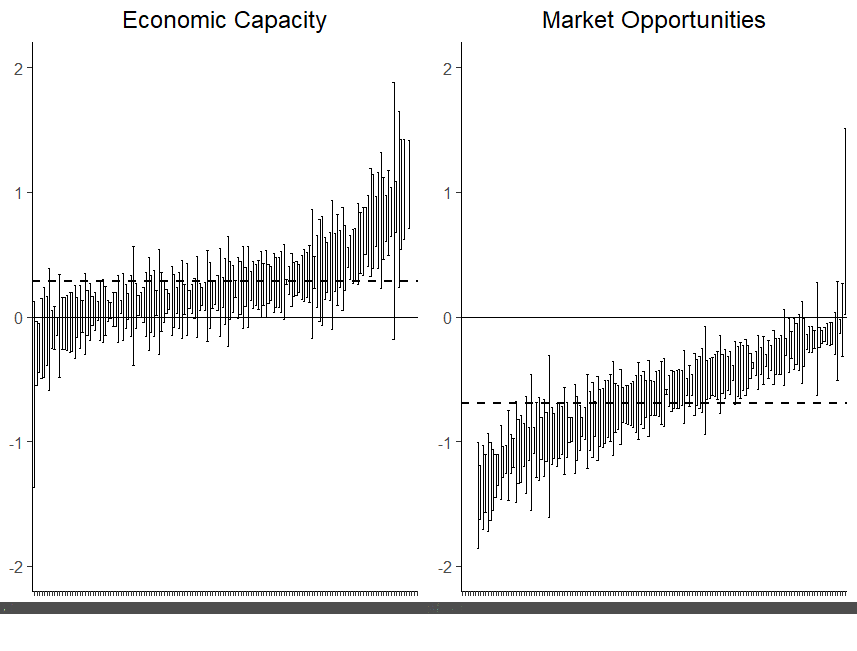
SM Figure 3. Estimates from the structural regression sub-model of the effects of latent variables on completed fertility. Error bars represent 95% confidence intervals. The y -axis is the effect size for a given survey, and the x-axis are the individual surveys, sorted from least to greatest in effect size.

**SENSITIVITY ANALYSES OF FIXED FACTOR LOADINGS**


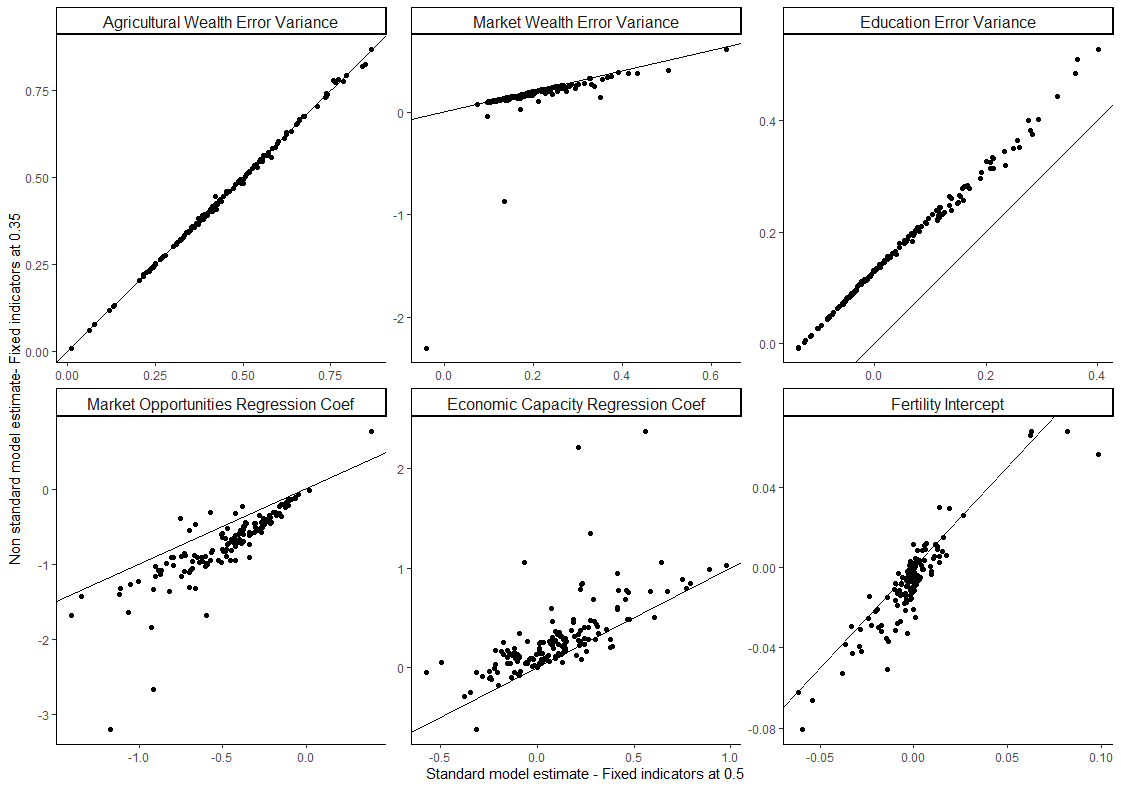


SM Figure 4. Results of Sensitivity Analysis.

The primary results of the sensitivity analyses are presented in SM Figure 4. Setting the fixed factor loading of education on market opportunities to the standard 0.5 resulted in a substantial number of the estimated error variances for education to be negative (Heyward cases; represented in the top row of Figure 4.). The top row of panels in SM Figure 4 represents the estimated measurement variance, with a line representing equality between the two model specifications. Rescaling the fixed factor loading for education on market opportunities from 0.5 to 0.35 uniformly shifted the estimated error variance for education by about +0.1, which resolved most of the negative variance cases. The re-scaling had little effects on the other estimated error parameters. (Top row of SM Figure 4).

Rescaling the latent variables by fixing the factor loading for education on market opportunities also resulted in minor changes to the distribution of the effects of the latent variables on fertility (Bottom row of SM Figure 4). The nonstandard models showed an increase in the absolute effect sizes for both main predictors in the structural regression (SM Figure 5).


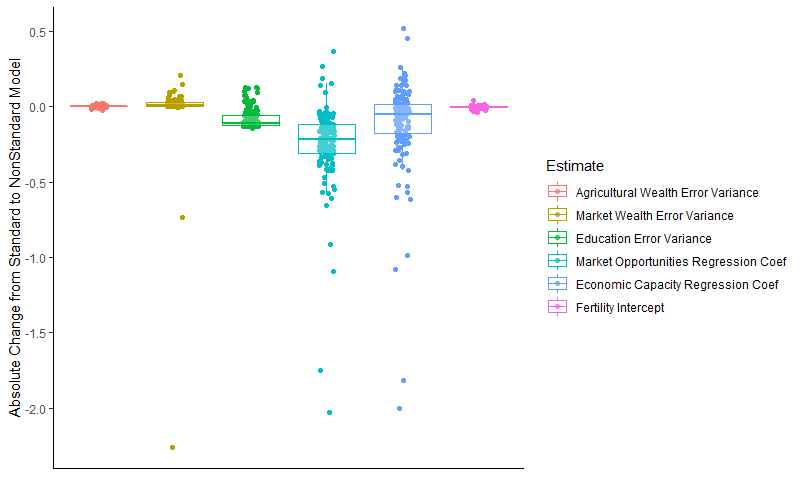


SM Figure 5. Absolute change in estimate size between standard and nonstandard multi-group models.

The absolute change in the regression coefficients for Economic Capacity and Market Opportunities show the greatest range, however for both the range is expanded due to large outlies that resulted from convergence issues within the standard model. SM Figure 4 bottom panels displays the relationship between the standard and non-standard models. Panel Market Opportunities Regression Coef, shows that despite the observed changes, the majority of the effects are still negative. Similarly, SM Figure 4 Panel Economic Capacity Regression Coef shows that despite the observed changes in effects across the standard and non-standard models, the effects are still largely positive.

**UNSTANDARDIZED MODEL OUTPUTS – FULL MODEL**

SM Table 3. Unstandardized model results for full sample.

|  | Full Sample Model |
| --- | --- |
|  | Estimate(Std.Err.) |
|  | Factor Loadings |
| Economic Capacity |  |
| Agricultural Wealth | 0.50^+^ |
| Market Wealth | 0.11(0.00)^***^ |
| Market Opportunities |  |
| Education | 0.50^+^ |
| Market Wealth | 0.21(0.00)^***^ |
|  | Regression Slopes |
| Fertility |  |
| Economic Capacity | 0.13(0.01)^***^ |
| Market Opportunities | -0.53(0.00)^***^ |
|  | Intercepts |
| Fertility | -0.00(0.00) |
| Agricultural Wealth | 0.00^+^ |
| Market Wealth | 0.00^+^ |
| Education | 0.00^+^ |
|  | Residual Variances |
| Agricultural Wealth | 0.41(0.00)^***^ |
| Market Wealth | 0.25(0.00)^***^ |
| Education | 0.14(0.00)^***^ |
| Fertility | 5.32(0.01)^***^ |
|  | Latent Variances |
| Economic Capacity | 1.00^+^ |
| Market Opportunities | 1.00^+^ |
|  | Latent Covariances |
| Economic Capacity w/Market Opportunities | 0.27(0.00)^***^ |
|  | Fit Indices |
| χ^2^ | 3295.97(4)^***^ |
| RMSEA | 0.04 |
| CFI | 0.97 |
| TLI | 0.96 |
| ^+^Fixed parameter | |
| ^*^p^**^p^***^p<0.001 | |

**UNSTANDARDIZED MODEL OUTPUTS – URBAN AND RURAL MODEL**

SM Table 4. Unstandardized model results for urban and rural sample.

|  | URBAN | RURAL |
| --- | --- | --- |
|  | Estimate(Std.Err.) | Estimate(Std.Err.) |
|  | Factor Loadings | |
| Economic Capacity |  |  |
| Agricultural Wealth | 0.50^+^ | 0.50^+^ |
| Market Wealth | 0.23(0.00)^***^ | 0.05(0.00)^***^ |
| Market Opportunities |  |  |
| Education | 0.50^+^ | 0.50^+^ |
| Market Wealth | 0.23(0.00)^***^ | 0.18(0.00)^***^ |
|  | Regression Slopes | |
| Fertility |  |  |
| Economic Capacity | 0.17(0.01)^***^ | 0.17(0.01)^***^ |
| Market Opportunities | -0.76(0.01)^***^ | -0.39(0.01)^***^ |
|  | Intercepts | |
| Fertility | 0.00(0.00) | -0.00(0.00) |
| Agricultural Wealth | 0.00^+^ | 0.00^+^ |
| Market Wealth | 0.00^+^ | 0.00^+^ |
| Education | 0.00^+^ | 0.00^+^ |
|  | Residual Variances | |
| Agricultural Wealth | 0.26(0.00)^***^ | 0.50(0.00)^***^ |
| Market Wealth | 0.22(0.00)^***^ | 0.25(0.00)^***^ |
| Education | 0.25(0.00)^***^ | 0.07(0.00)^***^ |
| Fertility | 4.16(0.01)^***^ | 5.97(0.01)^***^ |
|  | Latent Variances | |
| Economic Capacity | 1.00^+^ | 1.00^+^ |
| Market Opportunities | 1.00^+^ | 1.00^+^ |
|  | Latent Covariances | |
| Economic Capacity w/Market Opportunities | 0.38(0.00)^***^ | 0.21(0.00)^***^ |
|  | Fit Indices | |
| χ^2^ | 4509.14(8)^***^ |  |
| RMSEA | 0.04 |  |
| CFI | 0.96 |  |
| TLI | 0.94 |  |
| ^+^Fixed parameter | | |
| ^*^p^**^p^***^p<0.001 | | |


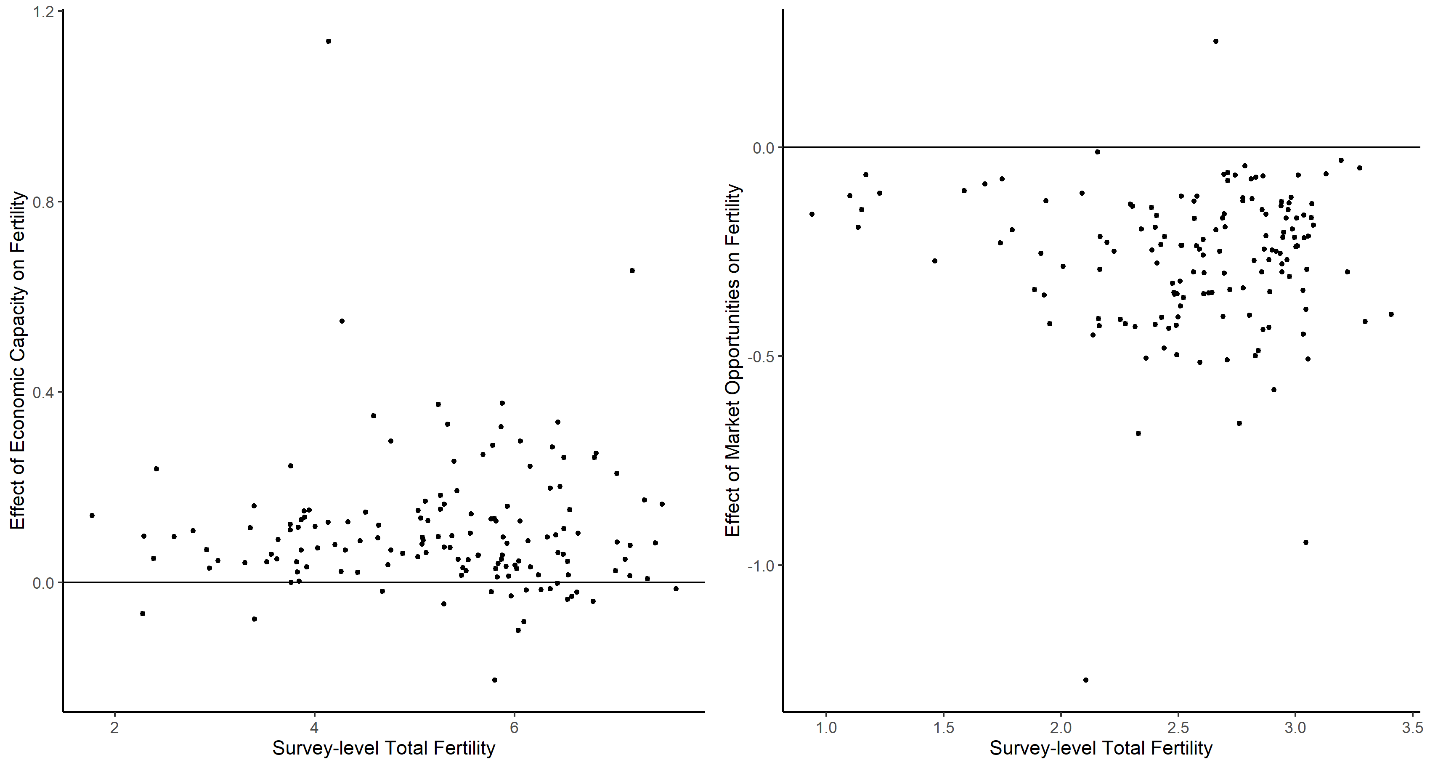


SM Figure 6. Distribution of Regression Effects on Fertility by Survey

**UNSTANDARDIZED MODEL OUTPUTS – MOST RECENT SURVEY WAVES ONLY**

SM Table 5. Unstandardized model results for just the most recent survey waves per country

|  | Full Recent Waves |
| --- | --- |
|  | Estimate(Std.Err.) |
|  | Factor Loadings |
| Economic Capacity |  |
| Agricultural Wealth | 0.50^+^ |
| Market Wealth | 0.13(0.00)^***^ |
| Market Opportunities |  |
| Education | 0.50^+^ |
| Market Wealth | 0.19(0.00)^***^ |
|  | Regression Slopes |
| Fertility |  |
| Economic Capacity | 0.11(0.01)^***^ |
| Market Opportunities | -0.49(0.01)^***^ |
|  | Intercepts |
| Fertility | -0.00(0.00) |
| Agricultural Wealth | 0.00^+^ |
| Market Wealth | 0.00^+^ |
| Education | 0.00^+^ |
|  | Residual Variances |
| Agricultural Wealth | 0.41(0.00)^***^ |
| Market Wealth | 0.23(0.00)^***^ |
| Education | 0.17(0.00)^***^ |
| Fertility | 4.49(0.01)^***^ |
|  | Latent Variances |
| Economic Capacity | 1.00^+^ |
| Market Opportunities | 1.00^+^ |
|  | Latent Covariances |
| Economic Capacity w/Market Opportunities | 0.28(0.00)^***^ |
|  | Fit Indices |
| χ^2^ | 1640.77(4)^***^ |
| RMSEA | 0.04 |
| CFI | 0.97 |
| TLI | 0.96 |
| ^+^Fixed parameter | |
| ^*^p^**^p^***^p<0.001 | |

**UNSTANDARDIZED MODEL OUTPUTS – MOST RECENT SURVEY WAVES ONLY STRATIFIED BY URBAN AND RURAL**

SM Table 6. Unstandardized model results for just the most recent survey waves per country, stratified by urban and rural

|  | Urban | Rural |
| --- | --- | --- |
|  | Estimate(Std.Err.) | Estimate(Std.Err.) |
|  | Factor Loadings | |
| Economic Capacity |  |  |
| Agricultural Wealth | 0.50^+^ | 0.50^+^ |
| Market Wealth | 0.09(0.00)^***^ | 0.22(0.00)^***^ |
| Market Opportunities |  |  |
| Education | 0.50^+^ | 0.50^+^ |
| Market Wealth | 0.19(0.00)^***^ | 0.18(0.00)^***^ |
|  | Regression Slopes | |
| Fertility |  |  |
| Economic Capacity | 0.12(0.01)^***^ | 0.13(0.01)^***^ |
| Market Opportunities | -0.39(0.01)^***^ | -0.65(0.01)^***^ |
|  | Intercepts | |
| Fertility | -0.00(0.01) | 0.00(0.01) |
| Agricultural Wealth | 0.00^+^ | 0.00^+^ |
| Market Wealth | 0.00^+^ | 0.00^+^ |
| Education | 0.00^+^ | 0.00^+^ |
|  | Residual Variances | |
| Agricultural Wealth | 0.51(0.00)^***^ | 0.23(0.00)^***^ |
| Market Wealth | 0.26(0.00)^***^ | 0.18(0.00)^***^ |
| Education | 0.12(0.00)^***^ | 0.24(0.00)^***^ |
| Fertility | 4.93(0.02)^***^ | 3.67(0.02)^***^ |
|  | Latent Variances | |
| Economic Capacity | 1.00^+^ | 1.00^+^ |
| Market Opportunities | 1.00^+^ | 1.00^+^ |
|  | Latent Covariances | |
| Economic Capacity w/Market Opportunities | 0.24(0.00)^***^ | 0.34(0.01)^***^ |
|  | Fit Indices | |
| χ^2^ | 1730.89(8)^***^ |  |
| RMSEA | 0.04 |  |
| CFI | 0.97 |  |
| TLI | 0.96 |  |
| ^+^Fixed parameter | | |
| ^*^p^**^p^***^p<0.001 | | |

SM Figure 7. Distribution of Fertility and Fertility Variance Across Surveys


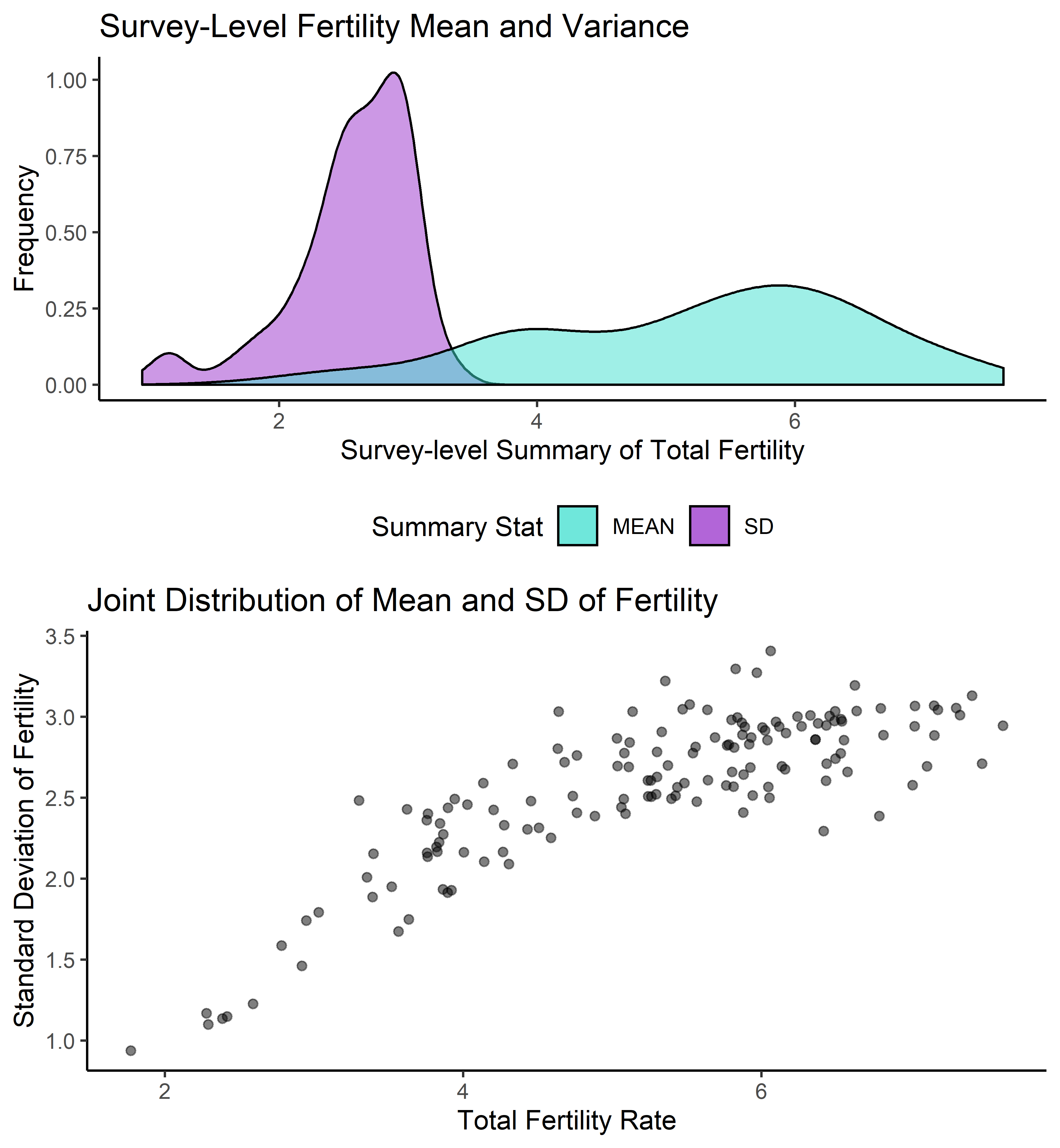


DESCRIPTION OF THE MCA VARIABLES

We used asset data typically used in the creation of the DHS wealth index, including information about household construction, assets, and access to basic services. (Hackman, Hruschka, and Vizireanu 2020). Coding nominal and count variables resulted in a total of 432 unique dichotomous variables across all surveys, ranging from 73 to 195 (see Appendix in (Hackman, Hruschka, and Vizireanu 2020) for full list of asset variables).

SM Figure 8. Distribution of Cronbach’s Alpha for the Agricultural Wealth Dimension.


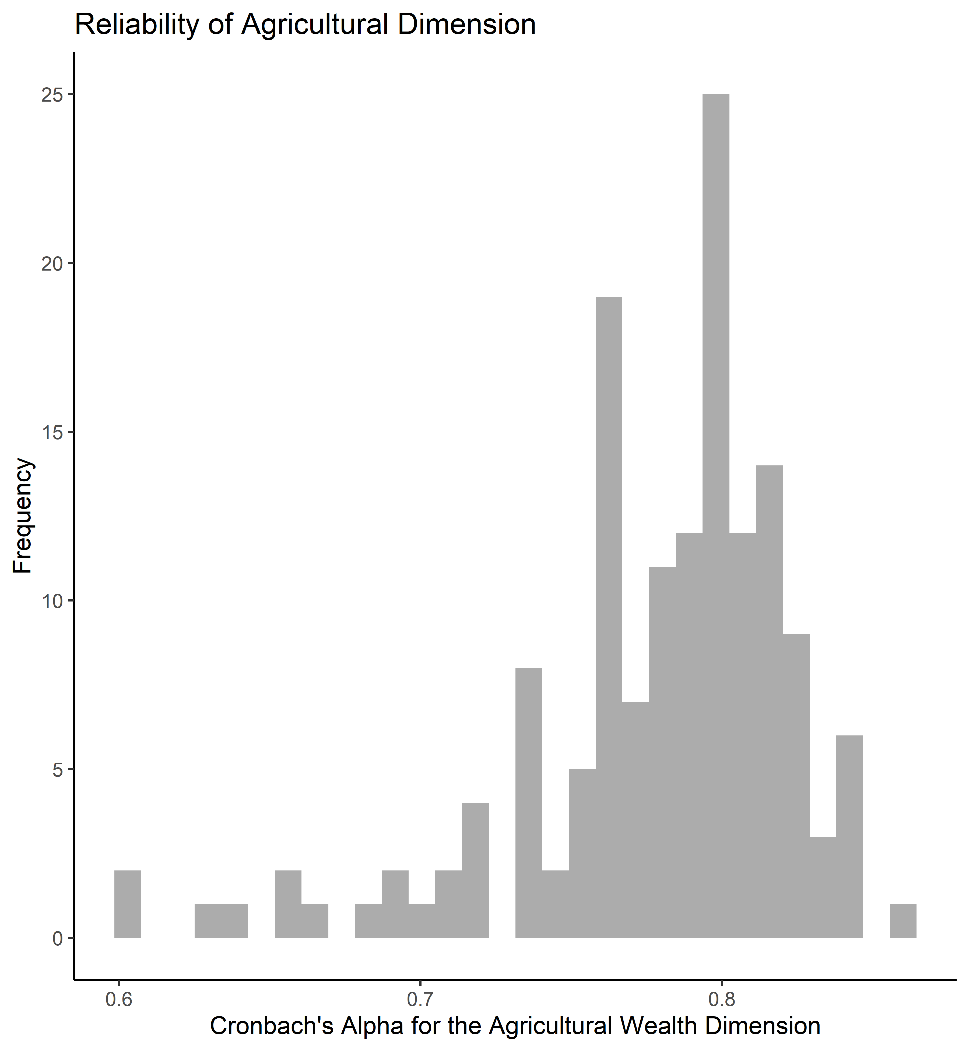


SM Table 7 – Effects of Economic Capacity and Market Opportunities on Fertility by Survey

|  |  | Effect of Economic Capacity on Fertility | | |  | Effect of Market Opportunities on Fertility | | |
| --- | --- | --- | --- | --- | --- | --- | --- | --- |
| Country | Survey | Unstandardized Coefficient | SE | P-Value |  | Unstandardized Coefficient | SE | P-Value |
| Afghanistan | AF 2015 | 0.05 | 0.02 | 0.01 |  | -0.06 | 0.01 | 0.00 |
| Afghanistan | AF 2010 | 0.07 | 0.02 | 0.00 |  | -0.14 | 0.02 | 0.00 |
| Albania | AL 2008 | 0.07 | 0.04 | 0.06 |  | -0.27 | 0.04 | 0.00 |
| Albania | AL 2017 | 0.05 | 0.03 | 0.11 |  | -0.19 | 0.03 | 0.00 |
| Armenia | AM 2016 | 0.10 | 0.04 | 0.03 |  | -0.12 | 0.04 | 0.00 |
| Armenia | AM 2005 | 0.10 | 0.04 | 0.01 |  | -0.11 | 0.02 | 0.00 |
| Armenia | AM 2010 | 0.24 | 0.05 | 0.00 |  | -0.15 | 0.04 | 0.00 |
| Angola | AO 2015 | 0.13 | 0.04 | 0.00 |  | -0.08 | 0.04 | 0.04 |
| Azerbaijan | AZ 2006 | 0.11 | 0.03 | 0.00 |  | -0.10 | 0.03 | 0.00 |
| Bangladesh | BD 2004 | 0.13 | 0.04 | 0.00 |  | -0.21 | 0.04 | 0.00 |
| Bangladesh | BD 1999 | 0.19 | 0.04 | 0.00 |  | -0.12 | 0.03 | 0.00 |
| Bangladesh | BD 2007 | 0.35 | 0.09 | 0.00 |  | -0.41 | 0.08 | 0.00 |
| Bangladesh | BD 2014 | 0.15 | 0.05 | 0.01 |  | -0.25 | 0.05 | 0.00 |
| Bangladesh | BD 2011 | 1.14 | 0.49 | 0.02 |  | -1.27 | 0.48 | 0.01 |
| Burkina Faso | BF 2018 | 0.10 | 0.05 | 0.03 |  | -0.14 | 0.03 | 0.00 |
| Burkina Faso | BF 2010 | -0.04 | 0.03 | 0.21 |  | -0.14 | 0.02 | 0.00 |
| Burkina Faso | BF 2014 | 0.02 | 0.05 | 0.63 |  | -0.12 | 0.03 | 0.00 |
| Benin | BJ 2012 | 0.10 | 0.04 | 0.01 |  | -0.22 | 0.03 | 0.00 |
| Benin | BJ 2017 | 0.01 | 0.04 | 0.74 |  | -0.23 | 0.03 | 0.00 |
| Benin | BJ 2006 | 0.00 | 0.03 | 0.95 |  | -0.26 | 0.03 | 0.00 |
| Burundi | BU 2010 | 0.11 | 0.04 | 0.00 |  | -0.07 | 0.03 | 0.04 |
| Burundi | BU 2016 | 0.24 | 0.03 | 0.00 |  | -0.25 | 0.03 | 0.00 |
| Burundi | BU 2012 | 0.34 | 0.07 | 0.00 |  | -0.08 | 0.05 | 0.12 |
| Congo Democratic Republic | CD 2013 | 0.20 | 0.05 | 0.00 |  | -0.17 | 0.04 | 0.00 |
| Congo | CG 2011 | 0.03 | 0.03 | 0.34 |  | -0.24 | 0.04 | 0.00 |
| Cote d'Ivoire | CI 2012 | 0.03 | 0.04 | 0.49 |  | -0.25 | 0.04 | 0.00 |
| Cameroon | CM 2011 | 0.04 | 0.03 | 0.23 |  | -0.22 | 0.04 | 0.00 |
| Dominican Republic | DR 2013 | 0.04 | 0.05 | 0.34 |  | -0.42 | 0.03 | 0.00 |
| Dominican Republic | DR 2007 | 0.12 | 0.03 | 0.00 |  | -0.41 | 0.02 | 0.00 |
| Egypt | EG 2008 | 0.02 | 0.02 | 0.26 |  | -0.14 | 0.02 | 0.00 |
| Egypt | EG 2014 | 0.07 | 0.02 | 0.00 |  | -0.13 | 0.02 | 0.00 |
| Egypt | EG 2005 | 0.04 | 0.02 | 0.11 |  | -0.23 | 0.02 | 0.00 |
| Ethiopia | ET 2016 | 0.33 | 0.04 | 0.00 |  | -0.27 | 0.03 | 0.00 |
| Ethiopia | ET 2011 | 0.26 | 0.03 | 0.00 |  | -0.16 | 0.03 | 0.00 |
| Ethiopia | ET 2005 | 0.28 | 0.03 | 0.00 |  | -0.17 | 0.03 | 0.00 |
| Gabon | GA 2012 | 0.03 | 0.04 | 0.55 |  | -0.19 | 0.04 | 0.00 |
| Ghana | GH 2008 | -0.05 | 0.07 | 0.51 |  | -0.36 | 0.06 | 0.00 |
| Ghana | GH 2014 | 0.09 | 0.05 | 0.07 |  | -0.42 | 0.03 | 0.00 |
| Ghana | GH 2016 | 0.08 | 0.05 | 0.13 |  | -0.43 | 0.05 | 0.00 |
| Gambia | GM 2013 | 0.09 | 0.06 | 0.15 |  | -0.30 | 0.05 | 0.00 |
| Guinea | GN 2012 | 0.04 | 0.04 | 0.31 |  | -0.13 | 0.04 | 0.00 |
| Guatemala | GU 2015 | 0.09 | 0.04 | 0.01 |  | -0.40 | 0.03 | 0.00 |
| Guyana | GY 2005 | -0.08 | 0.07 | 0.29 |  | -0.01 | 0.07 | 0.86 |
| Guyana | GY 2009 | 0.00 | 0.05 | 1.00 |  | -0.19 | 0.04 | 0.00 |
| Honduras | HN 2012 | -0.02 | 0.04 | 0.62 |  | -0.34 | 0.03 | 0.00 |
| Honduras | HN 2006 | 0.02 | 0.04 | 0.72 |  | -0.29 | 0.04 | 0.00 |
| Haiti | HT 2012 | 0.06 | 0.05 | 0.21 |  | -0.49 | 0.04 | 0.00 |
| Haiti | HT 2017 | 0.30 | 0.09 | 0.00 |  | -0.66 | 0.07 | 0.00 |
| Haiti | HT 2006 | 0.06 | 0.06 | 0.36 |  | -0.39 | 0.05 | 0.00 |
| India | IA 2006 | 0.24 | 0.02 | 0.00 |  | -0.45 | 0.02 | 0.00 |
| India | IA 1993 | 0.07 | 0.02 | 0.00 |  | -0.16 | 0.01 | 0.00 |
| India | IA 2015 | 0.16 | 0.01 | 0.00 |  | -0.34 | 0.01 | 0.00 |
| Indonesia | ID 2017 | 0.05 | 0.01 | 0.00 |  | -0.20 | 0.01 | 0.00 |
| Indonesia | ID 2007 | 0.02 | 0.02 | 0.24 |  | -0.21 | 0.01 | 0.00 |
| Indonesia | ID 2012 | 0.11 | 0.02 | 0.00 |  | -0.29 | 0.02 | 0.00 |
| Kenya | KE 2009 | 0.05 | 0.05 | 0.36 |  | -0.34 | 0.05 | 0.00 |
| Kenya | KE 2015 | 0.17 | 0.08 | 0.03 |  | -0.40 | 0.06 | 0.00 |
| Kenya | KE 2014 | 0.07 | 0.02 | 0.00 |  | -0.35 | 0.02 | 0.00 |
| Cambodia | KH 2010 | 0.08 | 0.03 | 0.01 |  | -0.23 | 0.03 | 0.00 |
| Cambodia | KH 2005 | 0.09 | 0.03 | 0.00 |  | -0.12 | 0.03 | 0.00 |
| Cambodia | KH 2014 | 0.12 | 0.04 | 0.00 |  | -0.25 | 0.04 | 0.00 |
| Comoros | KM 2012 | 0.13 | 0.08 | 0.12 |  | -0.45 | 0.06 | 0.00 |
| Kyrgyz Republic | KY 2012 | 0.06 | 0.03 | 0.03 |  | -0.09 | 0.03 | 0.00 |
| Liberia | LB 2007 | -0.01 | 0.05 | 0.79 |  | -0.07 | 0.06 | 0.22 |
| Liberia | LB 2013 | 0.15 | 0.06 | 0.01 |  | -0.30 | 0.06 | 0.00 |
| Liberia | LB 2016 | 0.26 | 0.16 | 0.10 |  | -0.51 | 0.14 | 0.00 |
| Liberia | LB 2011 | 0.65 | 0.50 | 0.19 |  | -0.95 | 0.51 | 0.06 |
| Lesotho | LS 2014 | 0.04 | 0.08 | 0.58 |  | -0.23 | 0.06 | 0.00 |
| Lesotho | LS 2009 | 0.55 | 0.17 | 0.00 |  | -0.68 | 0.15 | 0.00 |
| Morocco | MA 2003 | 0.12 | 0.03 | 0.00 |  | -0.34 | 0.03 | 0.00 |
| Moldova | MB 2005 | -0.07 | 0.03 | 0.04 |  | -0.07 | 0.02 | 0.00 |
| Madagascar | MD 2009 | 0.07 | 0.02 | 0.00 |  | -0.30 | 0.03 | 0.00 |
| Madagascar | MD 2016 | 0.05 | 0.05 | 0.33 |  | -0.24 | 0.04 | 0.00 |
| Mali | ML 2006 | 0.08 | 0.04 | 0.05 |  | -0.17 | 0.03 | 0.00 |
| Mali | ML 2001 | 0.08 | 0.04 | 0.03 |  | -0.06 | 0.03 | 0.01 |
| Mali | ML 2012 | 0.03 | 0.04 | 0.44 |  | -0.07 | 0.03 | 0.03 |
| Mali | ML 2015 | 0.01 | 0.05 | 0.88 |  | -0.07 | 0.04 | 0.07 |
| Myanmar | MM 2016 | 0.04 | 0.03 | 0.19 |  | -0.35 | 0.03 | 0.00 |
| Mauritania | MR 2003 | -0.03 | 0.07 | 0.69 |  | -0.05 | 0.06 | 0.45 |
| Malawi | MW 2014 | 0.03 | 0.08 | 0.72 |  | -0.17 | 0.07 | 0.02 |
| Malawi | MW 2010 | -0.03 | 0.03 | 0.32 |  | -0.20 | 0.03 | 0.00 |
| Malawi | MW 2012 | -0.21 | 0.12 | 0.10 |  | 0.25 | 0.12 | 0.04 |
| Malawi | MW 2017 | 0.14 | 0.09 | 0.09 |  | -0.33 | 0.07 | 0.00 |
| Malawi | MW 2004 | 0.04 | 0.05 | 0.34 |  | -0.13 | 0.04 | 0.00 |
| Malawi | MW 2015 | 0.06 | 0.03 | 0.07 |  | -0.28 | 0.03 | 0.00 |
| Mozambique | MZ 2018 | 0.27 | 0.07 | 0.00 |  | -0.16 | 0.06 | 0.00 |
| Mozambique | MZ 2011 | 0.16 | 0.04 | 0.00 |  | -0.04 | 0.03 | 0.12 |
| Mozambique | MZ 2015 | 0.10 | 0.05 | 0.03 |  | -0.12 | 0.04 | 0.00 |
| Nicaragua | NC 1998 | 0.01 | 0.03 | 0.74 |  | -0.42 | 0.03 | 0.00 |
| Nigeria | NG 2013 | 0.10 | 0.03 | 0.00 |  | -0.24 | 0.03 | 0.00 |
| Nigeria | NG 2008 | 0.10 | 0.02 | 0.00 |  | -0.22 | 0.02 | 0.00 |
| Nigeria | NG 2015 | 0.30 | 0.07 | 0.00 |  | -0.40 | 0.06 | 0.00 |
| Niger | NI 2012 | -0.01 | 0.04 | 0.74 |  | -0.22 | 0.04 | 0.00 |
| Namibia | NM 2007 | 0.09 | 0.05 | 0.07 |  | -0.35 | 0.04 | 0.00 |
| Namibia | NM 2013 | 0.13 | 0.05 | 0.01 |  | -0.42 | 0.04 | 0.00 |
| Nepal | NP 2016 | 0.03 | 0.04 | 0.40 |  | -0.35 | 0.04 | 0.00 |
| Nepal | NP 2006 | 0.06 | 0.03 | 0.02 |  | -0.25 | 0.03 | 0.00 |
| Nepal | NP 2011 | 0.02 | 0.03 | 0.44 |  | -0.29 | 0.03 | 0.00 |
| Peru | PE 2010 | 0.15 | 0.03 | 0.00 |  | -0.50 | 0.03 | 0.00 |
| Peru | PE 2012 | 0.11 | 0.03 | 0.00 |  | -0.50 | 0.02 | 0.00 |
| Peru | PE 2009 | 0.07 | 0.03 | 0.00 |  | -0.43 | 0.02 | 0.00 |
| Peru | PE 2006 | 0.13 | 0.04 | 0.00 |  | -0.51 | 0.03 | 0.00 |
| Peru | PE 2008 | 0.13 | 0.03 | 0.00 |  | -0.51 | 0.02 | 0.00 |
| Peru | PE 2011 | 0.14 | 0.03 | 0.00 |  | -0.48 | 0.02 | 0.00 |
| Philippines | PH 2017 | 0.05 | 0.03 | 0.15 |  | -0.41 | 0.03 | 0.00 |
| Pakistan | PK 2006 | 0.03 | 0.04 | 0.36 |  | -0.25 | 0.03 | 0.00 |
| Pakistan | PK 2012 | 0.29 | 0.06 | 0.00 |  | -0.50 | 0.06 | 0.00 |
| Pakistan | PK 2018 | 0.15 | 0.04 | 0.00 |  | -0.35 | 0.04 | 0.00 |
| Rwanda | RW 2013 | 0.13 | 0.08 | 0.08 |  | -0.24 | 0.06 | 0.00 |
| Rwanda | RW 2015 | 0.25 | 0.05 | 0.00 |  | -0.35 | 0.05 | 0.00 |
| Rwanda | RW 2010 | 0.38 | 0.06 | 0.00 |  | -0.35 | 0.06 | 0.00 |
| Rwanda | RW 2017 | 0.37 | 0.14 | 0.01 |  | -0.38 | 0.13 | 0.00 |
| Sierra Leone | SL 2016 | 0.06 | 0.07 | 0.36 |  | -0.20 | 0.05 | 0.00 |
| Sierra Leone | SL 2008 | 0.10 | 0.05 | 0.07 |  | -0.19 | 0.04 | 0.00 |
| Sierra Leone | SL 2013 | 0.08 | 0.04 | 0.02 |  | -0.17 | 0.03 | 0.00 |
| Senegal | SN 2005 | 0.02 | 0.03 | 0.64 |  | -0.13 | 0.03 | 0.00 |
| Senegal | SN 2008 | 0.02 | 0.03 | 0.56 |  | -0.24 | 0.03 | 0.00 |
| Senegal | SN 2010 | -0.08 | 0.03 | 0.01 |  | -0.15 | 0.03 | 0.00 |
| Senegal | SN 2012 | -0.02 | 0.04 | 0.72 |  | -0.28 | 0.04 | 0.00 |
| Senegal | SN 2014 | 0.04 | 0.04 | 0.42 |  | -0.25 | 0.04 | 0.00 |
| Senegal | SN 2015 | -0.10 | 0.05 | 0.03 |  | -0.15 | 0.04 | 0.00 |
| Senegal | SN 2016 | 0.05 | 0.06 | 0.38 |  | -0.35 | 0.05 | 0.00 |
| Senegal | SN 2017 | -0.02 | 0.04 | 0.62 |  | -0.27 | 0.04 | 0.00 |
| Senegal | SN 2006 | -0.04 | 0.05 | 0.43 |  | -0.20 | 0.04 | 0.00 |
| Sao Tome and Principe | ST 2008 | 0.13 | 0.10 | 0.19 |  | -0.41 | 0.07 | 0.00 |
| Swaziland | SZ 2006 | 0.33 | 0.15 | 0.03 |  | -0.58 | 0.13 | 0.00 |
| Chad | TD 2015 | 0.16 | 0.03 | 0.00 |  | -0.06 | 0.02 | 0.00 |
| Togo | TG 2014 | 0.06 | 0.05 | 0.24 |  | -0.30 | 0.04 | 0.00 |
| Togo | TG 2017 | 0.05 | 0.09 | 0.58 |  | -0.30 | 0.08 | 0.00 |
| Tajikistan | TJ 2017 | 0.09 | 0.03 | 0.00 |  | -0.08 | 0.03 | 0.00 |
| Tajikistan | TJ 2012 | 0.07 | 0.03 | 0.02 |  | -0.11 | 0.03 | 0.00 |
| Timor-Leste | TL 2009 | 0.10 | 0.03 | 0.01 |  | -0.14 | 0.03 | 0.00 |
| Timor-Leste | TL 2016 | 0.15 | 0.04 | 0.00 |  | -0.16 | 0.03 | 0.00 |
| Tanzania | TZ 2012 | -0.02 | 0.04 | 0.66 |  | -0.13 | 0.03 | 0.00 |
| Tanzania | TZ 2017 | 0.13 | 0.05 | 0.00 |  | -0.12 | 0.04 | 0.00 |
| Tanzania | TZ 2015 | 0.16 | 0.04 | 0.00 |  | -0.21 | 0.03 | 0.00 |
| Ukraine | UA 2007 | 0.14 | 0.04 | 0.00 |  | -0.16 | 0.03 | 0.00 |
| Uganda | UG 2006 | 0.17 | 0.05 | 0.00 |  | -0.21 | 0.04 | 0.00 |
| Uganda | UG 2011 | 0.01 | 0.04 | 0.70 |  | -0.14 | 0.04 | 0.00 |
| Uganda | UG 2014 | 0.08 | 0.06 | 0.16 |  | -0.27 | 0.06 | 0.00 |
| Uganda | UG 2016 | 0.27 | 0.06 | 0.00 |  | -0.43 | 0.05 | 0.00 |
| Uganda | UG 2009 | 0.23 | 0.08 | 0.00 |  | -0.30 | 0.08 | 0.00 |
| Yemen | YE 2013 | 0.24 | 0.03 | 0.00 |  | -0.19 | 0.03 | 0.00 |
| South Africa | ZA 1998 | 0.00 | 0.03 | 0.92 |  | -0.20 | 0.03 | 0.00 |
| South Africa | ZA 2016 | 0.03 | 0.04 | 0.40 |  | -0.23 | 0.03 | 0.00 |
| Zambia | ZM 2007 | 0.06 | 0.05 | 0.27 |  | -0.31 | 0.06 | 0.00 |
| Zambia | ZM 2013 | 0.20 | 0.04 | 0.00 |  | -0.44 | 0.04 | 0.00 |
| Zimbabwe | ZW 1988 | -0.02 | 0.06 | 0.74 |  | -0.03 | 0.06 | 0.59 |
| Zimbabwe | ZW 2005 | 0.18 | 0.05 | 0.00 |  | -0.32 | 0.05 | 0.00 |
| Zimbabwe | ZW 2010 | 0.15 | 0.06 | 0.01 |  | -0.43 | 0.05 | 0.00 |
| Zimbabwe | ZW 2015 | 0.12 | 0.06 | 0.05 |  | -0.43 | 0.05 | 0.00 |

The primary models in the manuscript stratify the sample either by survey wave or by urban and rural contexts. This approach collapses important variation both within countries across urban and rural contexts, and broadly assumes that urban and rural contexts are roughly similar across the sample. In many of the survey waves data limitations prevented stratifying by urban and rural contexts within a country for a given survey year. Only 70% (106 out of 151) of the surveys converged without negative variances. For models that did converge, the effects were qualitatively similar to those that did not stratify by urban-rural for a given survey. That is, economic capacity maintained a positive effect on fertility, while market opportunities held a consistent negative effect on fertility (SM Figure 10). We hesitate to comment on the interpretation of the variation observed in the stratified estimates, compared to the full model estimates. The differences could be due to variation in resource and market opportunity gradients across urban and rural contexts, or increased noise in running models on smaller sample sizes.


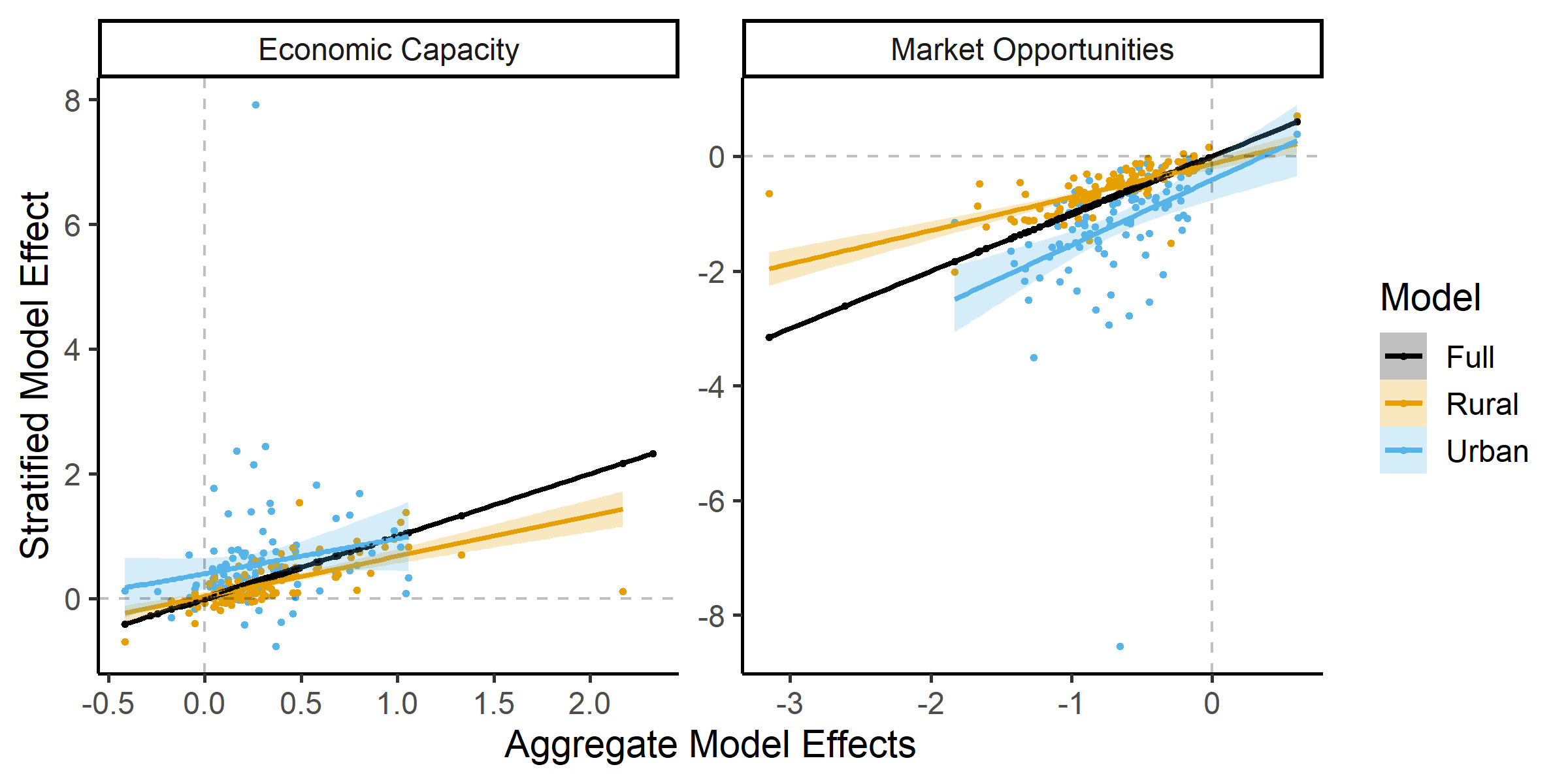


SM Figure 10. Latent variable effects on fertility when stratifying by survey and urban/ rural contexts. The full model (black line), represents equality. In the models stratified by urban-rural, the effects were generally larger in urban contexts than estimated in the full model.

**UNSTANDARDIZED MODEL OUTPUTS – URBAN SENSITIVITY ANALYSIS**

SM Table 8. Unstandardized model results for the urban sub-sample excluding households that are poor in agricultural wealth and those that are both poor in agricultural wealth and rich in market-based wealth.

|  | Urban Excluding Poor Agricultural Wealth | Urban Excluding Poor Agricultural Wealth and High Market Wealth |
| --- | --- | --- |
|  | Estimate(Std.Err.) | Estimate(Std.Err.) |
|  | Factor Loadings | |
| Economic Capacity |  |  |
| Agricultural Wealth | 0.50^+^ | 0.50^+^ |
| Market Wealth | 0.17(0.00)^***^ | 0.24(0.00)^***^ |
| Market Opportunities |  |  |
| Education | 0.50^+^ | 0.50^+^ |
| Market Wealth | 0.27(0.00)^***^ | 0.23(0.00)^***^ |
|  | Regression Slopes | |
| Fertility |  |  |
| Economic Capacity | 0.14(0.01)^***^ | 0.16(0.01)^***^ |
| Market Opportunities | -0.76(0.01)^***^ | -0.76(0.01)^***^ |
|  | Intercepts | |
| Fertility | 0.02(0.01)^***^ | 0.01(0.00) |
| Agricultural Wealth | 0.00^+^ | 0.00^+^ |
| Market Wealth | 0.00^+^ | 0.00^+^ |
| Education | 0.00^+^ | 0.00^+^ |
|  | Residual Variances | |
| Agricultural Wealth | 0.27(0.00)^***^ | 0.27(0.00)^***^ |
| Market Wealth | 0.24(0.00)^***^ | 0.22(0.00)^***^ |
| Education | 0.26(0.00)^***^ | 0.25(0.00)^***^ |
| Fertility | 3.87(0.02)^***^ | 4.13(0.02)^***^ |
|  | Latent Variances | |
| Economic Capacity | 1.00^+^ | 1.00^+^ |
| Market Opportunities | 1.00^+^ | 1.00^+^ |
|  | Latent Covariances | |
| Economic Capacity w/Market Opportunities | 0.34(0.01)^***^ | 0.40(0.00)^***^ |
|  | Fit Indices | |
| χ^2^ | 26705.92(4)^***^ | 2225.33(4)^***^ |
| RMSEA | 0.22 | 0.05 |
| CFI | 0.29 | 0.97 |
| TLI | -0.07 | 0.95 |
| ^+^Fixed parameter | | |
| ^*^p^**^p^***^p<0.001 | | |

SM REFERENCES

Hackman, Joseph, Daniel Hruschka, and Mariya Vizireanu. 2020. “An Agricultural Wealth Index for Multidimensional Wealth Assessments.” *Population and Development Review*, October, padr.12367. https://doi.org/10.1111/padr.12367.
